# Papillary renal neoplasm with reverse polarity: a novel entity for the next WHO classification? a clinical-pathological and molecular study of 15 cases, compared to eosinophilic/oncocytic papillary renal cell carcinoma

**DOI:** 10.1007/s00428-026-04536-9

**Published:** 2026-04-29

**Authors:** Stefano Marletta, Denise Fiorini, Anna Caliò, Lisa Marcolini, Lavinia Stefanizzi, Cinzia Giacometti, Serena Pedron, Caterina Vicentini, Guido Martignoni

**Affiliations:** 1 Department of Biomedical Science, Humanitas University, Milan, Italy; 2 Division of Pathology, Humanitas Istituto Clinico Catanese, Catania, Italy; 3 Department of Pathology and Laboratory Medicine, Pederzoli Hospital, Peschiera del Garda, Italy; 4 Department of Diagnostic and Public Health, Section of Pathology, University of Verona, Largo L. Scuro 10, 37134 Verona, Italy

**Keywords:** Papillary renal neoplasm with reverse polarity, Eosinophilic/oncocytic papillary renal cell carcinoma, Oncocytic renal tumors, Differential diagnosis

## Abstract

Papillary renal neoplasm with reverse polarity (PRNRP) is a recently recognized renal tumor characterized by papillary architecture lined by a single layer of low-grade eosinophilic cells with apically located nuclei. In the latest WHO classification, they are not recognized as a distinct entity but rather as a morphological pattern of papillary renal cell carcinoma. To date, limited studies have compared PRNRP with eosinophilic/oncocytic papillary renal cell carcinoma (E/OPRCC), which they are not infrequently confused with, although a few previous comparisons with classic papillary renal cell carcinoma have already identified several distinguishing features. A comparative analysis of 15 of PRNRPs and 16 of E/OPRCC cases was conducted, evaluating their histopathological, immunophenotypic, interphase cytogenetic, and molecular profiles. PRNRPs demonstrated distinctive morphological features, including consistent apical nuclear positioning, absence of foamy macrophages in the papillary cores (*p* = 0.0001), and a lower nucleolar grade compared to E/OPRCCs (p = < 0.0001). Immunohistochemically, PRNRPs exhibited strong and uniform GATA3 expression and were negative for vimentin, CD10, and CD13, in contrast to E/OPRCCs. Cytogenetically, PRNRPs lacked trisomies of chromosomes 7 and 17, which were present in ~ 40% of E/OPRCCs (p = 0.0262). Notably, all PRNRP cases harbored *KRAS* mutations, absent in E/OPRCCs (*p* < 0.0001). Our findings support the classification of PRNRP as a distinct renal tumor entity, separated from both classic papillary renal cell carcinoma and its eosinophilic/oncocytic morphological variant.

**Supplementary Information:**

The online version contains supplementary material available at https://doi.org/10.1007/s00428-026-04536-9.

## Introduction

Papillary renal cell carcinoma is the second most common renal cancer following clear cell renal carcinoma, accounting for 15–20% of all forms of kidney cancer[1]. Firstly described in 1976[2], since 1997, papillary renal cell carcinomas have been further divided into two subtypes according to their morphological features[3], Type 1 and Type 2. This classification seemed to carry prognostic implications, as Type 2 papillary renal cell carcinomas often showed lymphovascular invasion, recurrences, nodal and distant metastases, and, definitely, a poorer outcome[4]. However, tumors with different or overlapping features between the two subtypes not fulfilling the diagnostic criteria for each category have been variously described, such as oncocytic papillary tumors exhibiting distinct cytogenetic alterations [5–7]. Additionally, a proportion of the cases included in the Type 2 category belonged to specific molecularly defined subsets, such as fumarate hydratase (FH)-deficient and TFE3-rearranged renal cell carcinoma[8]. In order to address these discrepancies and to integrate them with the evolving histological and molecular insights into renal cell carcinomas, a novel subtyping system for papillary renal cell carcinoma was proposed in 2017, based on immunohistochemical and molecular findings[9]. According to this latter, Type 4 papillary renal cell carcinomas were defined as tumors made up of oncocytic cells with an apical orientation in a linear manner, no evidence of nuclear pseudostratification, and positive GATA3 staining at immunohistochemical analysis.

A subset of oncocytic papillary renal cell carcinomas showing inverted nuclear polarity was first reported in 2009[10], but it was not definitely recognized as a distinct renal cell neoplasm until 2019, when a series of 18 cases of papillary renal cell carcinomas with peculiar morphological and molecular hallmarks was described[11]. The Authors named these tumors “papillary renal neoplasm with reverse polarity (PRNRP)”. Their morphological and immunohistochemical findings suggested a differentiation toward the distal nephron and were overall overlapping with the 2017 Type 4 category [9] and smaller oncocytic tumors classified as Type D adenomas [12]. The term “neoplasm” instead of “carcinoma” was preferred as all the studied lesions were small in size and behaved in a benign fashion. The same authors later reported a high frequency of *KRAS* mutations in their own series of tumors, a relatively uncommon finding among other types of papillary renal cell carcinomas and, in general, among renal cell carcinomas[13, 14]. Similar cases were subsequently observed[15–17], all showing peculiar histological features, a high rate of *KRAS* mutations, compared to papillary renal cell carcinoma[18, 19], and an indolent clinical behavior. To date, PRNRP is included as a morphological pattern of papillary renal cell carcinoma in the 2022 WHO classification[20].

Indeed, the main differential diagnosis of PRNRP is represented by papillary renal cell carcinomas with extensive eosinophilic features. In this view, tumors with a papillary architecture, oncocytoma-like cytology, and chromosomal alterations peculiar to papillary renal cell carcinoma (i.e., 7 and 17 trisomies, Y losses) have been known since 2003[6], and generally termed as “eosinophilic/oncocytic papillary renal cell carcinomas (E/OPRCC)”[7]. Although less aggressive than classic Type 2 papillary renal cell carcinomas, such neoplasms still carry a low but well-defined malignant potential, and ought to be distinguished from PRNRP[6, 19, 21]. However, comprehensive clinical-pathological and molecular comparison between these two tumors remains limited. Herein, we report a series of thirty‑two renal cell neoplasms with papillary architecture and oncocytic cytology, aiming to identify their distinctive clinical, morphological, immunophenotypical, and molecular features. The goal of this study was to classify each neoplasm as PRNRP or E/OPRCC, contributing to a more precise nosographic definition of these tumors.

## Methods

### Patients and samples

Thirty-two unreported renal cell neoplasms with papillary architecture and eosinophilic/oncocytic cytology were retrieved from the files of the institutions (Pederzoli Hospital and University of Verona, Italy). All slides were reviewed by three pathologists with a recognized uropathology background (SM, AC, and GM). For each case, the following morphologic features were recorded: architecture, peritumoral pseudocapsule, coagulative tumor necrosis, cholesterol clefts, foamy macrophages, intraepithelial hemosiderin pigment, psammomatous bodies, and hyalinization of papillae stroma. Nucleolar grade according to ISUP/WHO 2022[20] was assessed. All procedures performed involving human participants received approval (Prog. 4136CESC) and were in accordance with the ethical standards of the institutional and/or national research committee, as well as the Declaration of Helsinki.

### Immunohistochemistry

Sections from tissue blocks from each case were immunohistochemically stained with the following antibodies: CD10 (clone 56C6, dilution 1:50; Novocastra), alpha-methylacyl-CoA racemase AMACR (clone 13H7, dilution 1:25; Dako), CD13 (clone 38C12, dilution 1:100; Novocastra), cytokeratin (CK) 7 (clone RN7, dilution 1:100; Novocastra), MET (clone SP44, prediluted; Ventana), parvalbumin (clone P19, dilution 1:500; Sigma), carbonic anhydrase 9 (polyclonal rabbit, dilution 1:1000; Abcam), cathepsin K (clone 3F9, dilution 1:2000; Abcam), CK 34βE12 (clone 34βE12, dilution 1:100; Leica), GATA3 (clone L50-823, dilution 1:150; BD Pharmingen), S100A1 (clone M01, dilution 1:600; Abnova), BRAF (clone V6003, prediluted; Roche), E-cadherin (clone 36, prediluted; Roche), CK AE1/AE3 (clone AE1 & AE3 ratio 20:1, dilution 1/100; Leica), EMA (clone GP 1.4, dilution 1/100; Leica), CD117 (clone T595, dilution 1/100; Leica), vimentin (clone V9, dilution 1/400; Leica), MUC1 (clone 695, prediluted; Biocare), Ki67 (clone K2, prediluted; Leica), PAX8 (clone MRQ-50, prediluted; Roche) and WT1 (clone WT49, dilution 1/20; Leica).

All samples were processed using a sensitive Bond Polymer Refine detection system in an automated Bond immunohistochemistry instrument (Leica Biosystems, Germany). MET, BRAF, E-cadherin, and PAX8 immunohistochemical analyses were performed using Ventana Benchmark XT. The appropriate positive and negative controls were concurrently carried out. Labeling for each marker was recorded as the percentage of positive cells.

### Fluorescence *in situ* hybridization (FISH)

FISH analysis was carried out to detect numerical chromosomal abnormalities involving chromosomes 7, 17, and Y, using centromere-specific probes: CEP7 (D7Z1, Leica), CEP17 (D17Z1, Leica), and CEPY (DYZ3, ZytoVision GmbH).

In brief, 3-µm sections were obtained from formalin-fixed paraffin-embedded (FFPE) tissue blocks and mounted on positively charged slides. Slides were dried for 1 h at 60 °C, then deparaffinized, rehydrated, and fixed in methanol/acetic acid (3:1) for 5 min. Pretreatment consisted of incubation at 85 °C for 30 min in 0.1 M citrate buffer (pH 6), followed by pepsin digestion (4 mg/mL in 0.9% NaCl, pH 1.5) for 8 min at 37 °C. After washing and dehydration, 10 µL of probe was applied to the target area and sealed with rubber cement. Denaturation was performed by incubating slides at 80 °C for 10 min in a humidified chamber (Thermobrite System), followed by overnight hybridization at 37 °C. After removing the rubber cement and coverslip, slides were washed in 2 × SSC/0.3% NP-40 for 15 min at room temperature and then at 72 °C for 2 min. Sections were counterstained with DAPI antifade (ProLong Gold Antifade Reagent; Life Technologies, USA) and examined under an Olympus BX61 fluorescence microscope (Olympus, Japan) equipped with appropriate filter sets, using a × 60–100 oil immersion objective.

A minimum of 100 non-overlapping neoplastic nuclei were scored. Trisomy of chromosomes 7 and 17 was defined as ≥ 3 signals in more than 20% of tumor cells, while loss of the Y chromosome was considered significant when ≥ 45% of tumor cells lacked the signal as previously reported[22]. Results were compared with adjacent non-tumoral renal parenchyma.

### KRAS mutational status

Mutations of the *KRAS* gene were analyzed using an Idylla™ platform (Biocartis, Mechelen, Belgium) using the Idylla KRAS Mutation Test cartridges (Biocartis). Idylla is a fully cartridge-based automated quantitative PCR platform and uses microfluidics processing with all reagents on board. For each analysis, three sections were macrodissected and then transferred to a wetted (nuclease-free water) filter paper according to the manufacturer’s instructions. A second wetted filter paper was then added on top of the FFPE material, and the sample with the two wetted filter papers was finally placed on the lysis pad in the Idylla cartridge and inserted in the instrument. Inside the cartridge, the sample was homogenized, and cells were lysed using a combination of high-intensity-focused ultrasound, enzymatic/chemical digestion, and heat. The nucleic acids were liberated and ready for subsequent polymerase chain reaction (PCR) amplification. The PCR was performed using a real-time fluorophore-based detection system. After a 130-min run, all steps were automatically performed inside the cartridge, and final reports were directly available on the system after an automatic on-board post-PCR curve analysis. Total *KRAS* was used as a sample processing control.

### Literature review and statistical analysis

A review was conducted on the PubMed database up to January 2026 to identify any case series or report regarding PRNRP and to compare them with the present cohort. The search strategy was carried out using the following keywords: *oncocytic papillary renal cell carcinoma, oncocytic PRCC, papillary renal neoplasm with reverse polarity,* or *PRNRP.* Reviews were excluded, as were studies dealing with other cancer types and those on animal or in vitro models/cell cultures. The χ^2^ test and Fisher's exact test were used to compare categorical data for clinical, pathological, immunohistochemical, and molecular findings, while the Student *t*-test was used to compare continuous data. All p-values were based on a 2-sided hypothesis, and the results were considered statistically significant if < 0.05.

## Results

### Clinical characteristics

The clinical findings of the present series are detailed in Table 1. Based on the observed morphologic features, 15 cases were classified as PRNRP and 16 as E/OPRCC. Among the whole ten-year renal cell tumor cohort, PRNRP accounted for 0.54% of the neoplasms (15/2110) and about 3% of all papillary renal cell carcinomas. As for the PRNRP patients, 11 were male and 4 were female (F:M ratio 1:2.75) and aged from 52 to 86 years old (mean and median 70), whereas in the E/OPRCC group all but one patient were males (F:M ratio 1:15), with the age at diagnosis spanning from 54 to 79 years old (mean 67, median 64). Most tumors (24/31, 77%) were removed by partial nephrectomy (12/15 PRNRPs, 80%, and 12/16 E/OPRCCs, 75%), whereas the remaining patients (7/31, 23%) underwent radical nephrectomy (3/15 PRNRPs, 20%, and 4/16 E/OPRCCs, 25%). The mean tumor size for PRNRPs was 1.4 cm, ranging from 0.4 to 3.2 cm; therefore, they were all staged as pT1a according to the 8th edition of the AJCC Cancer Staging Manual[23]. Conversely, the E/OPRCC lesions were on average 3.4 cm in size, spanning from 0.4 to 10 cm; thus, 11 cases were staged as pT1a, 2 cases as pT1b, and 3 cases as pT3a. Two patients presented with synchronous lesions, namely one subject with two PRNRPs and an E/OPRCC (PRNRP cases 7–8 and E/OPRCC case 6), while another was diagnosed with one neoplasm each (PRNRP case 11 and E/OPRCC case 5).

**Table 1:** Clinical characteristics of the tumors from the present series

Case n	Histotype	Age	Sex	Size/laterality	TNM stage	Surgery	Follow up (months)	Other findings
1	PRNRP	52	M	1.2 cm/L	pT1aNxMx	PN	3 NED	None
2	PRNRP	79	M	2 cm/R	pT1aNxMx	PN	9 NED	None
3	PRNRP	71	M	2.2 cm/L	pT1aNxMx	PN	72 NED	None
4	PRNRP	69	M	0.7 cm/R	pT1aNxMx	PN	124 NED	PRCC
5	PRNRP	71	M	2 cm/L	pT1aNxMx	PN	58 NED	Renal angiomyolipoma
6	PRNRP	79	M	3.2 cm/R	pT1aNxMx	PN	NA	PRCC
7	PRNRP*	77	M	0.8 cm/R	pT1aNxMx	RN	44 NED	E/OPRCC case 6, renal oncocytoma
8	PRNRP*	77	M	3 cm/L	pT1aNxMx	PN	44 NED	E/OPRCC case 6, renal oncocytoma
9	PRNRP	62	M	0.4 cm/R	pT1aNxMx	RN	58 NED	CCRCC lung metastases
10	PRNRP	66	M	0.4 cm/L	pT1aNxMx	PN	1 NED	CCRCC
11	PRNRP^#^	62	F	0.5 cm/R	pT1aNxMx	PN	2 NED	E/OPRCC case 5
12	PRNRP	76	F	2.3 cm/R	pT1aNxMx	PN	NA	None
13	PRNRP	66	F	1 cm/R	pT1aNxMx	PN	21 NED	Borderline mucinous ovarian tumor
14	PRNRP	52	M	1.1 cm/R	pT1aNxMx	PN	23 NED	None
15	PRNRP	86	F	0.5 cm/L	pT1aNxMx	RN	29 NED	None
1	E/OPRCC	60	M	2.2 cm/R	pT1aNxMx	PN	NA	None
2	E/OPRCC	60	M	1.2 cm/L	pT1aNxMx	PN	93 NED	None
3	E/OPRCC	64	M	6 cm/R	pT1bNxMx	PN	3 NED	None
4	E/OPRCC	64	M	2.6 cm/R	pT1aNxMx	PN	44 NED	None
5	E/OPRCC^#^	62	F	4.5 cm/R	pT1bNxMx	PN	2 NED	PRNRP case 11
6	E/OPRCC*	77	M	10 cm/R	pT3aNxMx	RN	44 NED	PRNRP cases 7 and 8
7	E/OPRCC	79	M	5.5 cm/L	pT3aN1M1	PN	68 AWD	PRCC nodal metastases
8	E/OPRCC	77	M	0.4 cm/L	pT1aNxMx	PN	22 NED	None
9	E/OPRCC	73	M	3 cm/L	pT1aNxMx	RN	44 NED	CCRCC
10	E/OPRCC	54	M	2 cm/L	pT1aNxMx	PN	2 NED	None
11	E/OPRCC	62	M	5 cm/L	pT3aN0Mx	RN	8 NED	None
12	E/OPRCC	78	M	1 cm/R	pT1aNxMx	RN	71 NED	None
13	E/OPRCC	69	M	3.5 cm/R	pT1aNxMx	PN	76 NED	CCRCC
14	E/OPRCC	68	M	2.5 cm/L	pT1aNxMx	PN	1 NED	None
15	E/OPRCC	63	M	2.2 cm/L	pT1aNxMx	PN	4 NED	None
16	E/OPRCC	65	M	3 cm/R	pT1aNxMx	PN	119 NED	None

^*^,^#^ same patient

Abbreviations: *PRNRP* Papillary renal neoplasm with reverse polarity, *M* Male, *L* left, *PN* Partial nephrectomy, *NED* No evidence of disease, *R* Right, *PRCC* Papillary renal cell carcinoma, *NA* Not available, *RN* Radical nephrectomy, *E/OPRCC* Eosinophilic/oncocytic papillary renal cell carcinoma, *CCRCC* Clear cell renal cell carcinoma, *F* Female, *AWD* Alive with disease

Follow-up was available for 29 patients, ranging from 1 to 124 months (mean 39, median 36). To date, all PRNRP patients are alive with no evidence of recurrent or metastatic disease. One patient (case 9) was found with histologically documented pulmonary metastases from a synchronous ipsilateral clear cell renal cell carcinoma. In the E/OPRCC group, a millimetric lesion of uncertain clinical significance at the site of prior renal partial resection was radiologically detected in one patient (case 4) 44 months after surgery and remains on follow‑up. Another patient (case 7) developed histologically confirmed papillary renal cell carcinoma lymph node metastases along with lung localizations.

### Pathological features

**Fig. 1:**
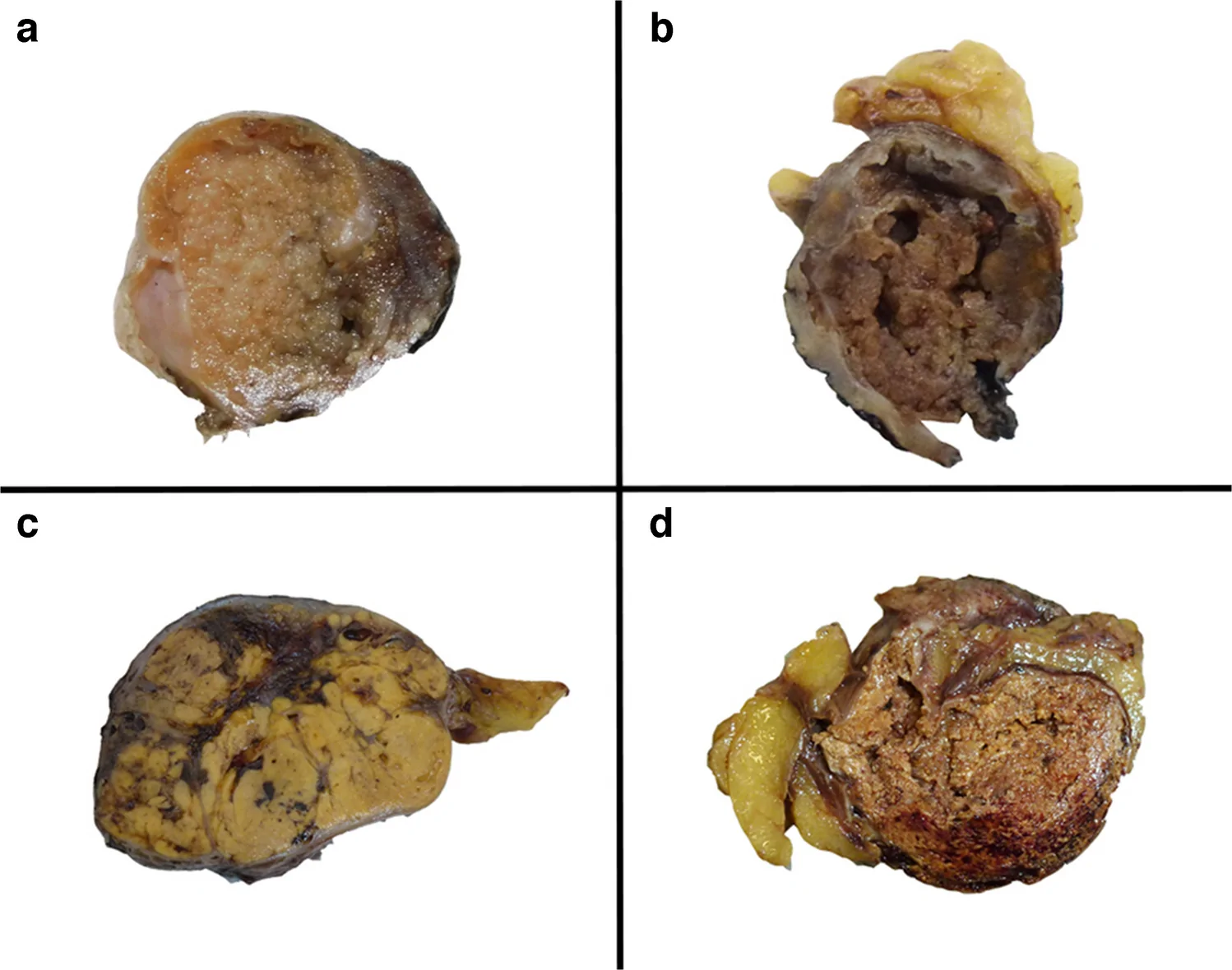
Gross photographs from PRNRP, cases 5 (**a**) and 3 (**b**), revealing gray-brownish granular lesions, and E/OPRCC, cases 8 (**c**) and 13 (**d**), which instead were frequently yellowish in color, due to their enrichment in foamy macrophages

**Fig. 2:**
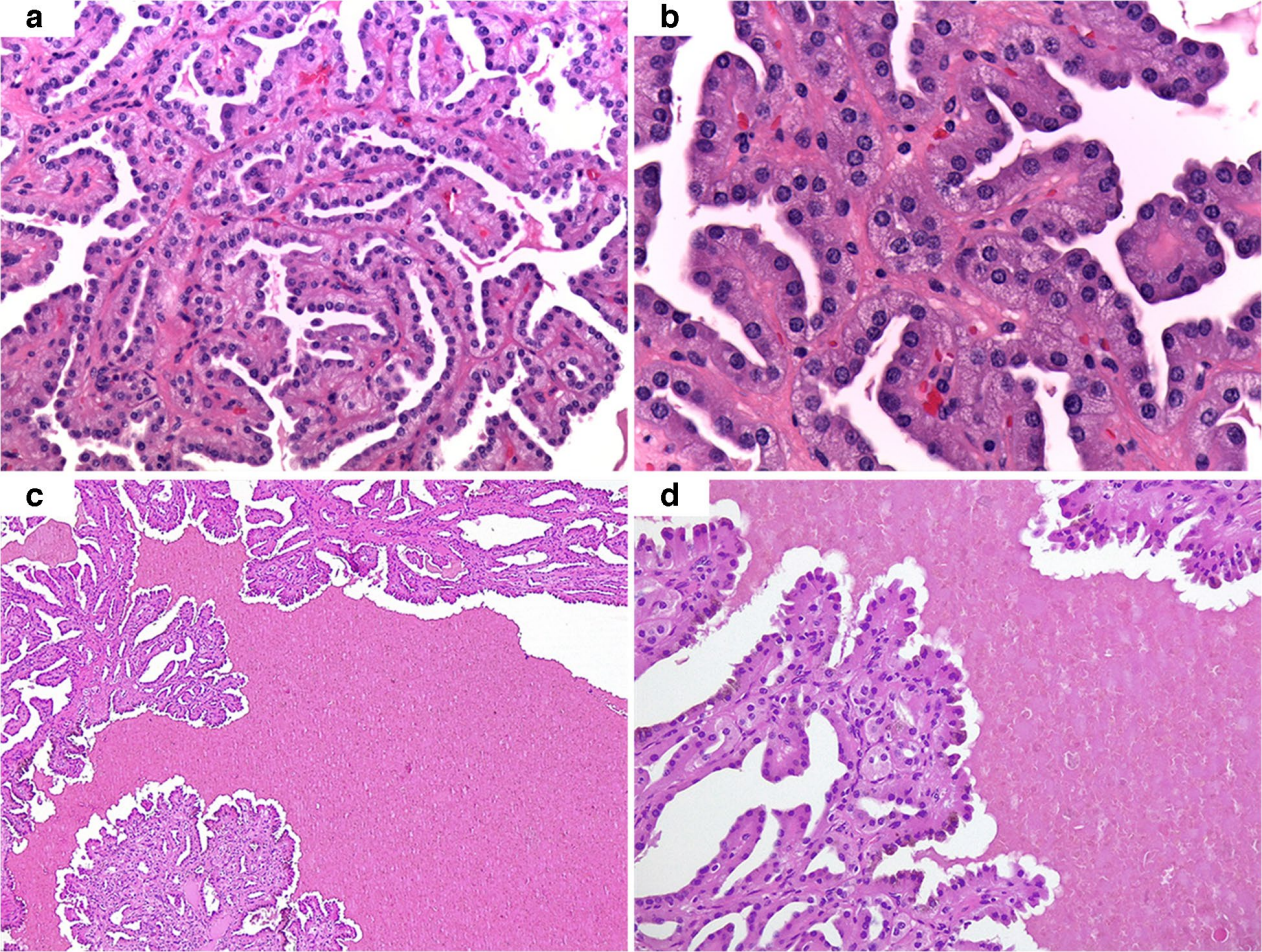
Morphological features of PRNRP. Tumors made up of branched papillae, supported by a frequently hyalinized stroma, with a “mosaic-like” pattern (**a**), lined by oncocytic cells with apically oriented nuclei and inconspicuous nucleoli (ISUP/WHO G1) (**b**). A cystic appearance filled with amorphous eosinophilic material was not uncommon (**c**). Scattered foamy macrophages were only occasionally detected (**d**) [original magnifications 50X c, 200X a and c, and 400X b]

From a macroscopic standpoint, PRNRPs displayed a brownish to grayish color, a finely granular cut surface, and expansile borders (Figs. 1A-B). Microscopically, they were often well circumscribed (12/15, 80%) and surrounded by a fibrous pseudocapsule, which was occasionally discontinuous in 5 cases. The tumors were composed predominantly of thin, branching, “mosaic‑like” papillary structures with fibrovascular cores, sometimes containing hyalinized stroma (Fig. 2A). The papillae were lined by a single layer of columnar‑to‑cuboidal epithelial cells with finely granular eosinophilic cytoplasm. Characteristically, the nuclei were located at the apical surface or luminal side of the cells, away from the basement membrane (“reverse polarity”), and were mostly small, not overlapping, and with inconspicuous nucleoli (Fig. 2B). Hence, according to ISUP/WHO grading criteria, all PRNRP were either graded as G1 (10/15, 67%) or G2 (5/15, 33%). A cystic component was sometimes identified (5/15, 33%), occasionally representing the dominant pattern, with lumens filled with amorphous eosinophilic material containing isolated cholesterol clefts (4/15, 27%) (Fig. 2C). Foamy macrophages were only rarely observed within the papillary stroma (2/15, 13%) (Fig. 2D), and intraepithelial hemosiderin deposition was noted in only one case (1/15, 7%). Neither necrosis nor psammoma bodies were identified in any of the cases (0/15).

**Fig. 3:**
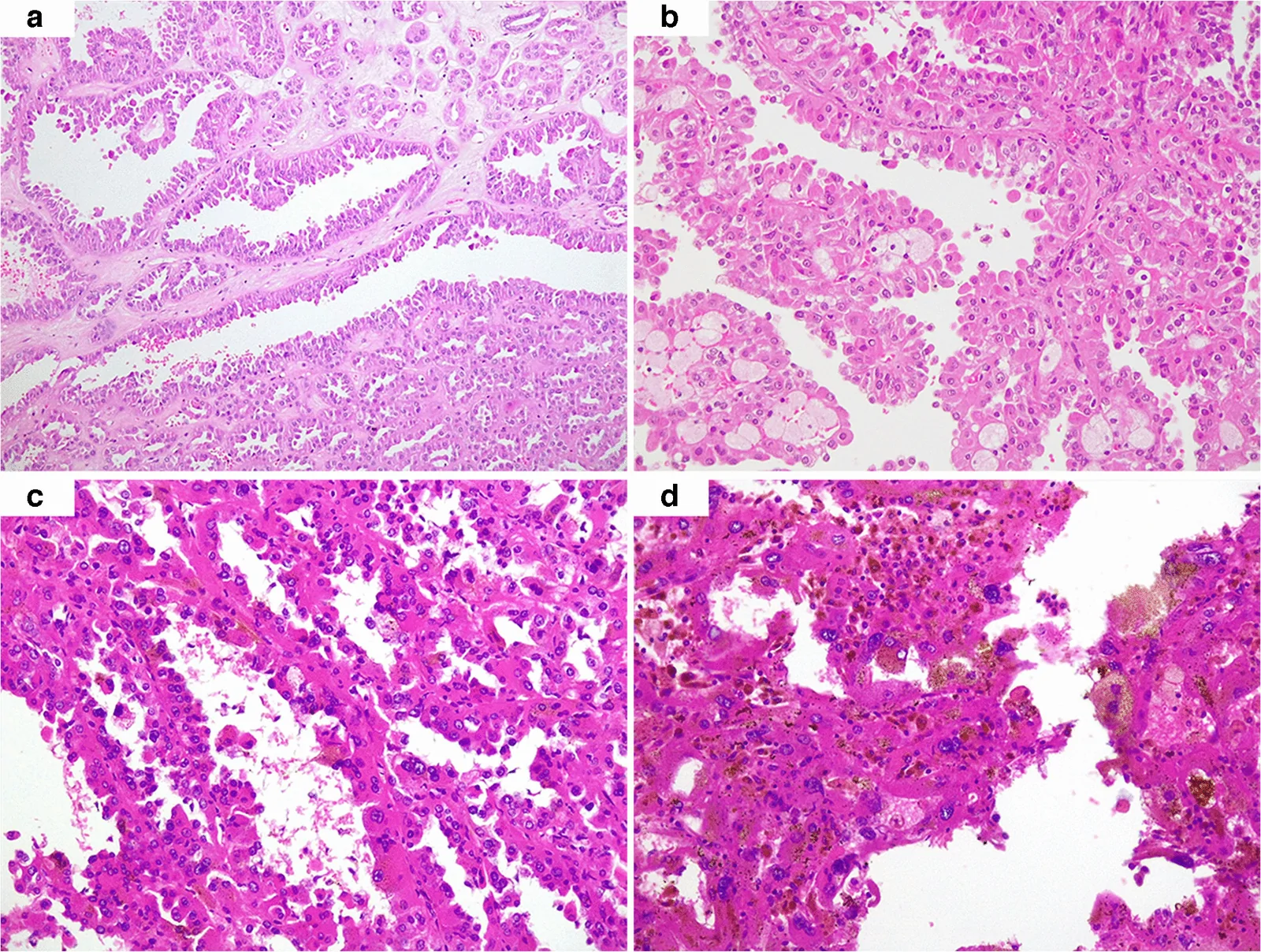
Histological characters of E/OPRCC. Compared to PRNPR, E/OPRCC often displayed a tubular/solid architecture close to papillary areas (**a**). Foamy macrophages (**b**) and hemosiderin pigment (**c**) were frequently observed. More than half of the cases showed high-grade nuclear features (ISUP/WHO G3) (**d**)

As far as E/OPRCCs were concerned, they exhibited a greater heterogeneity at gross examination, typically showing a predominantly solid‑compact though sometimes friable‑granular appearance, ranging from yellowish to brown‑gray in color (Figs. 1C-D). Morphologically, in addition to the papillary pattern, a tubular/solid architecture was observed in slightly a third of the cases (6/16, 37%) (Fig. 3A). A fibrous and often discontinuous pseudocapsule was detected in most tumors (12/16, 75%), while cholesterol clefts were found in a proportion of the specimens (5/16, 31%). Foamy macrophages and intraepithelial hemosiderin pigment were observed in more than half (10/16, 62%) and one‑third (6/16, 37%) of the samples (Figs. 3B-C), respectively. Papillary hyalinization was rare (3/16, 19%) and, when present, very focal. Tumor coagulative necrosis was documented in two cases (12%), as well as psammoma bodies in five tumors (31%). Regarding ISUP/WHO nucleolar grading, one case was classified as G1 (6%), six as G2 (37%), and nine as G3 (56%) (Fig. 3D).

The pathological features are summarized in Table 2.

**Table 2:** Pathological features of PRNRPs and E/OPRCCs from the present series

Case n	Histotype	ISUP/WHO grading	Architecture	Pseudocapsule	Necrosis	Hemosiderin pigment	Cholesterol clefts	Foamy macrophages	Psammomatous bodies	Stromal hyalinization	Inflammatory infiltrate
1	PRNRP	1	Papillary (100%)	Discontinuous	Absent	Absent	Absent	Absent	Absent	Present (F)	Present
2	PRNRP	2	Papillary (70%), cystic (30%)	Continuous	Absent	Present (F)	Present	Absent	Absent	Present (F)	Present
3	PRNRP	2	Papillary (100%)	Continuous	Absent	Absent	Absent	Absent	Absent	Absent	Absent
4	PRNRP	2	Papillary (100%)	Discontinuous	Absent	Absent	Absent	Absent	Absent	Absent	Absent
5	PRNRP	2	Papillary (100%)	Continuous	Absent	Absent	Absent	Absent	Absent	Present	Present
6	PRNRP	2	Papillary (100%)	Discontinuous	Absent	Absent	Absent	Absent	Absent	Present (F)	Absent
7	PRNRP	1	Papillary (70%), cystic (30%)	Continuous	Absent	Absent	Absent	Absent	Absent	Present (F)	Absent
8	PRNRP	1	Papillary (90%), cystic (10%)	Continuous	Absent	Absent	Present	Absent	Absent	Present (F)	Present
9	PRNRP	1	Papillary (100%)	Continuous	Absent	Absent	Present	Absent	Absent	Absent	Absent
10	PRNRP	1	Papillary (100%)	Discontinuous	Absent	Absent	Absent	Absent	Absent	Absent	Present
11	PRNRP	1	Papillary (100%)	Discontinuous	Absent	Absent	Absent	Absent	Absent	Absent	Present
12	PRNRP	1	Papillary (40%), cystic (60%)	Continuous	Absent	Absent	Present	Present (F)	Absent	Present (F)	Absent
13	PRNRP	1	Papillary (100%)	Absent	Absent	Absent	Absent	Absent	Absent	Absent	Absent
14	PRNRP	1	Papillary (90%), cystic (10%)	Continuous	Absent	Absent	Present	Present (F)	Absent	Present	Present
15	PRNRP	1	Papillary (100%)	Absent	Absent	Absent	Absent	Absent	Absent	Present	Absent
1	E/OPRCC	2	Papillary (10%), tubular (90%)	Absent	Absent	Absent	Absent	Absent	Present (F)	Present	Absent
2	E/OPRCC	3	Papillary (100%)	Discontinuous	Absent	Absent	Present	Present	Present (F)	Absent	Present
3	E/OPRCC	3	Papillary (100%)	Discontinuous	Absent	Absent	Absent	Present	Present (F)	Absent	Absent
4	E/OPRCC	2	Papillary (100%)	Discontinuous	Absent	Present	Absent	Present	Absent	Absent	Present
5	E/OPRCC	1	Papillary (100%)	Discontinuous	Absent	Absent	Present	Present	Absent	Absent	Absent
6	E/OPRCC	2	Papillary (70%), cystic (30%)	Discontinuous	Absent	Present	Absent	Absent	Absent	Absent	Absent
7	E/OPRCC	3	Papillary (10%), tubular (90%)	Discontinuous	Present	Present	Absent	Absent	Absent	F	Absent
8	E/OPRCC	2	Papillary (100%)	Discontinuous	Absent	Absent	Absent	Present	Absent	Absent	Absent
9	E/OPRCC	2	Papillary (10%), tubular (90%)	Absent	Absent	Present	Present	Absent	Absent	Absent	Present
10	E/OPRCC	2	Papillary (5%), tubular (95%)	Absent	Absent	Absent	Absent	Present	Present	F	Absent
11	E/OPRCC	3	Papillary (10%), tubular (90%)	Absent	Present (F)	Absent	Absent	Absent	Absent	Absent	Absent
12	E/OPRCC	3	Papillary (5%), tubular (95%)	Continuous	Absent	Present	Absent	Absent	Absent	Absent	Absent
13	E/OPRCC	3	Papillary (100%)	Continuous	Absent	Present	Present	Present	Present	Absent	Present
14	E/OPRCC	3	Papillary (100%)	Continuous	Absent	Absent	Absent	Present	Absent	Absent	Absent
15	E/OPRCC	3	Papillary (100%)	Discontinuous	Absent	Absent	Present	Present	Absent	Absent	Present
16	E/OPRCC	3	Papillary (100%)	Continuous	Absent	Absent	Absent	Present	Absent	Absent	Absent

Abbreviations: *PRNRP* Papillary renal neoplasm with reverse polarity, *F* Focal, *E/OPRCC* Eosinophilic/oncocytic papillary renal cell carcinoma

### Immunohistochemical findings (Fig. 4)

**Fig. 4:**
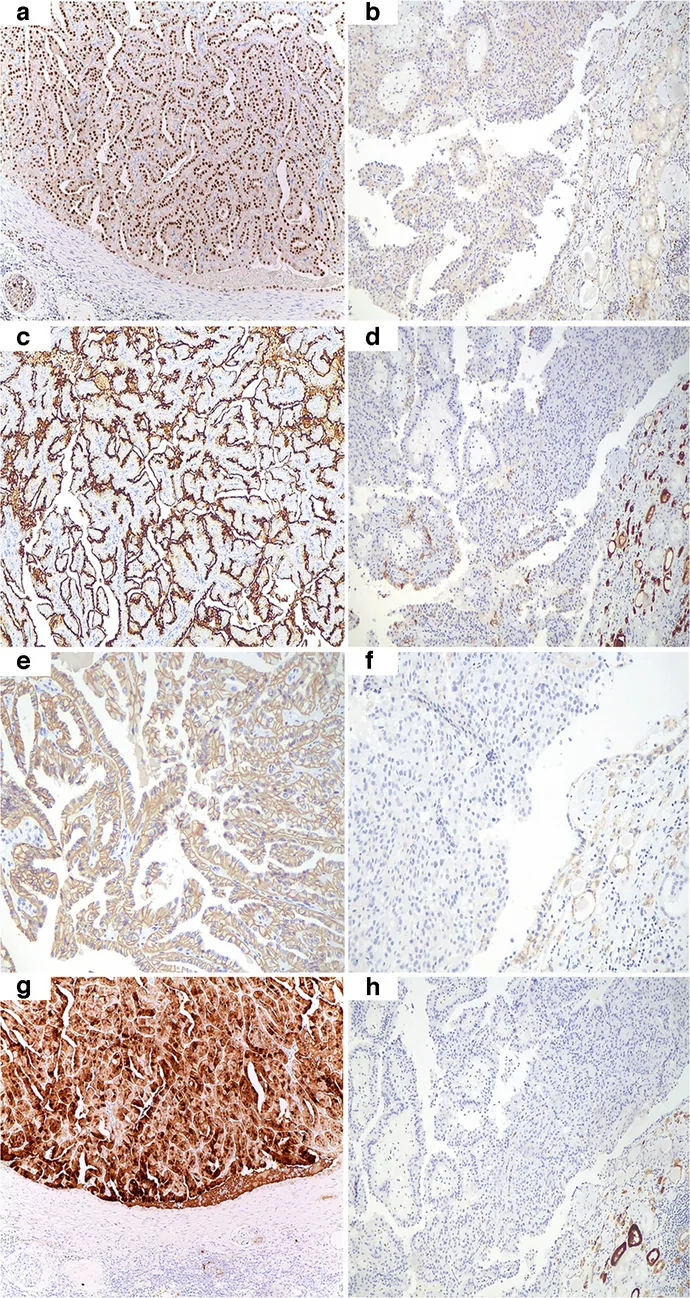
Immunohistochemical comparison of PRNRP and E/OPRCC. While the former was generally positive for GATA3 (a), MUC1 (c), E-cadherin (e), and parvalbumin (g), the latter usually labeled negative for these markers (b, d, f, h; normal renal tubules in the adjacent parenchyma worked as positive internal control) [original magnifications 100X a, b, c, d, g, and h, and 200X e, f]

All PRNRP cases demonstrated a consistent immunophenotypic profile characterized by diffuse expression of PAX8, MUC1, AE1/AE3, CK7, and GATA3. In contrast, they uniformly lacked expression of CD13, vimentin, CD117, carbonic anhydrase 9, cathepsin K, BRAF, and WT1. Most tumors also showed strong EMA staining (87%), with only a single exception, and frequently labeled for E‑cadherin (75%), CK34βE12 (75%), MET (75%), and parvalbumin (64%), although with variable intensity. S100A1 and AMACR were strongly positive in approximately half of the cases (55% and 44%, respectively), while CD10 expression was rarely detected (25%) and generally with weak to moderate staining. Proliferative activity was consistently low, with a mean Ki‑67 index below 1%.

On the other hand, E/OPRCCs exhibited a substantially different immunophenotype. These tumors were uniformly negative for carbonic anhydrase 9, cathepsin K, CD117, WT1, and BRAF, while being uniformly positive for AMACR (100%) and largely for CD10 (87%). GATA3 staining was constantly absent (0%). Most tumors exhibited significant expression of the proximal tubular marker CD13 (87%), as well as MET (81%) and vimentin (67%), and variable expression of EMA (40%). Similarly, S100A1 tended to show strong staining in the majority of the neoplasms tested (69%). CK7 and AE1/AE3 were expressed in about half of the cases, while MUC1 was less consistently detected (33%). CK34βE12 was rarely positive (20%), and distal tubular marker parvalbumin showed weak to moderate labeling in only a minority of the investigated samples (14%). E‑cadherin expression, when present, was typically strong but limited to a very small subset of lesions (7%). Like PRNRP, E/OPRCCs revealed a low Ki67 proliferative index, averaging around 1.3%.

The immunohistochemical results of the 15 PRNRPs and 16 E/OPRCCs are tabulated in Tables 3 and 4, respectively.

**Table 3:** Immunohistochemical findings of PRNRPs from the present series

Case n	1	2	3	4	5	6	7	8	9	10	11	12	13	14	15
**Histotype**	**PRNRP**	**PRNRP**	**PRNRP**	**PRNRP**	**PRNRP**	**PRNRP**	**PRNRP**	**PRNRP**	**PRNRP**	**PRNRP**	**PRNRP**	**PRNRP**	**PRNRP**	**PRNRP**	**PRNRP**
**CK7**	**80%**	**90%**	**90%**	**70%**	**80%**	**NA**	**NA**	**NA**	**NA**	**90%**	**70%**	**80%**	**90%**	**90%**	**NA**
CAIX	Neg	Neg	Neg	Neg	Neg	NA	NA	NA	NA	NA	Neg	Neg	Neg	Neg	Neg
**GATA3**	**90%**	**70%**	**80%**	**30% F**	**90%**	**NA**	**NA**	**NA**	**90%**	**90%**	**90%**	**90%**	**90%**	**90%**	**90%**
PAX8	90%	70%	80%	40% F	80%	NA	NA	NA	NA	NA	90%	NA	90%	80%	NA
**E-cadherin**	**Neg**	**30% F**	**90%**	**90%**	**90%**	**NA**	**NA**	**NA**	**NA**	**NA**	**40%**	**NA**	**Neg**	**30%**	**NA**
**EMA**	**Neg**	**90%**	**90%**	**90%**	**90%**	**NA**	**NA**	**NA**	**NA**	**NA**	**80%**	**NA**	**90%**	**90%**	**NA**
**CD10**	**Neg**	**60%**	**Neg**	**Neg**	**Neg**	**NA**	**NA**	**NA**	**NA**	**NA**	**80%**	**NA**	**Neg**	**Neg**	**NA**
CD117	Neg	Neg	Neg	Neg	Neg	NA	NA	NA	NA	NA	Neg	Neg	Neg	Neg	NA
**CKAE1-E3**	**70%**	**90%**	**90%**	**80%**	**80%**	**NA**	**NA**	**NA**	**NA**	**NA**	**90%**	**NA**	**90%**	**90%**	**NA**
**CK34βE12**	**Neg**	**70%**	**70%**	**Neg**	**70%**	**NA**	**NA**	**NA**	**NA**	**NA**	**90%**	**NA**	**90%**	**90%**	**NA**
**Vimentin**	**Neg**	**Neg**	**Neg**	**Neg**	**Neg**	**NA**	**NA**	**NA**	**NA**	**NA**	**Neg**	**Neg**	**NA**	**Neg**	**NA**
**AMACR**	**Neg**	**Neg**	**70%**	**Neg**	**70%**	**NA**	**NA**	**NA**	**NA**	**NA**	**90%**	**NA**	**90%**	**Neg**	**Neg**
**CD13**	**Neg**	**Neg**	**Neg**	**Neg**	**Neg**	**NA**	**NA**	**NA**	**NA**	**NA**	**Neg**	**NA**	**Neg**	**Neg**	**NA**
MET	Neg	30% F	80%	Neg	80%	NA	NA	NA	NA	NA	80%	NA	90%	60%	NA
**PV**	**Neg**	**70%**	**70%**	**Neg**	**80%**	**NA**	**NA**	**NA**	**90%**	**80%**	**Neg**	**80%**	**Neg**	**90%**	**NA**
Cathepsin K	Neg	Neg	Neg	Neg	Neg	NA	NA	NA	NA	NA	Neg	NA	Neg	Neg	NA
S100A1	Neg	90%	90%	90%	Neg	NA	NA	NA	NA	NA	Neg	80%	Neg	80%	NA
BRAF	Neg	Neg	Neg	Neg	Neg	NA	NA	NA	NA	NA	Neg	NA	Neg	Neg	NA
**MUC1**	**80%**	**90%**	**90%**	**80%**	**90%**	**NA**	**NA**	**NA**	**NA**	**NA**	**20% F**	**NA**	**90%**	**90%**	**NA**
Ki67	2%	1%	1%	1%	< 1%	NA	NA	NA	NA	NA	1%	NA	< 1%	< 1%	NA
WT1	Neg	Neg	Neg	Neg	Neg	NA	NA	NA	NA	NA	Neg	NA	Neg	Neg	NA

Abbreviations: *PRNRP* Papillary renal neoplasm with reverse polarity, *CK* Cytokeratin, *Neg.* Negative, *NA* Not available, *CAIX* Carbonic anhydrase IX, *F* Focal, *AMACR* Alpha-methylacyl-CoA racemase, *PV* Parvalbumin

**Table 4:** Immunohistochemical findings of E/OPRCCs from the present series

Case n	1	2	3	4	5	6	7	8	9	10	11	12	13	14	15	16
**Histotype**	**E/OPRCC**	**E/OPRCC**	**E/OPRCC**	**E/OPRCC**	**E/OPRCC**	**E/OPRCC**	**E/OPRCC**	**E/OPRCC**	**E/OPRCC**	**E/OPRCC**	**E/OPRCC**	**E/OPRCC**	**E/OPRCC**	**E/OPRCC**	**E/OPRCC**	**E/OPRCC**
**CK7**	**20% F**	**Neg**	**Neg**	**Neg**	**90%**	**NA**	**10%**	**70%**	**90%**	**80%**	**Neg**	**Neg**	**Neg**	**90%**	**Neg**	**Neg**
CAIX	Neg	Neg	Neg	Neg	Neg	NA	Neg	Neg	Neg	Neg	Neg	Neg	Neg	Neg	Neg	Neg
**GATA3**	**Neg**	**Neg**	**Neg**	**Neg**	**Neg**	**NA**	**Neg**	**Neg**	**Neg**	**Neg**	**Neg**	**Neg**	**Neg**	**Neg**	**Neg**	**Neg**
PAX8	70%	Neg	Neg	70%	90%	NA	70%	90%	Neg	90%	Neg	NA	70%	90%	90%	80%
**E-cadherin**	**Neg**	**NA**	**Neg**	**Neg**	**Neg**	**NA**	**Neg**	**Neg**	**Neg**	**Neg**	**Neg**	**Neg**	**Neg**	**Neg**	**Neg**	**60%**
**EMA**	**30% F**	**80%**	**Neg**	**Neg**	**90%**	**NA**	**30% F**	**70%**	**Neg**	**50%**	**Neg**	**Neg**	**Neg**	**30% F**	**50%**	**90%**
**CD10**	**90%**	**80%**	**80%**	**90%**	**50%**	**NA**	**90%**	**90%**	**20% F**	**60%**	**60%**	**Neg**	**90%**	**70%**	**90%**	**80%**
CD117	Neg	Neg	Neg	Neg	Neg	NA	Neg	Neg	Neg	Neg	Neg	Neg	Neg	Neg	Neg	Neg
**CKAE1-AE3**	**Neg**	**Neg**	**Neg**	**60%**	**90%**	**NA**	**Neg**	**70%**	**Neg**	**70%**	**50%**	**Neg**	**50%**	**90%**	**Neg**	**Neg**
**CK34βE12**	**Neg**	**Neg**	**Neg**	**Neg**	**50%**	**NA**	**Neg**	**Neg**	**Neg**	**20% F**	**Neg**	**Neg**	**Neg**	**50%**	**Neg**	**Neg**
**Vimentin**	**Neg**	**Neg**	**30% F**	**30% F**	**Neg**	**NA**	**80%**	**Neg**	**60%**	**80%**	**20% F**	**Neg**	**20% F**	**20% F**	**80%**	**70%**
**AMACR**	**60%**	**50%**	**90%**	**90%**	**60%**	**NA**	**90%**	**20% F**	**90%**	**90%**	**90%**	**30% F**	**90%**	**90%**	**90%**	**70%**
**CD13**	**90%**	**Neg**	**90%**	**90%**	**Neg**	**NA**	**90%**	**90%**	**70%**	**70%**	**70%**	**10%**	**90%**	**10%**	**90%**	**80%**
MET	60%	60%	60%	60%	90%	NA	30% F	80%	20% F	60%	Neg	Neg	60%	20% F	80%	Neg
**PV**	**Neg**	**NA**	**Neg**	**50%**	**30% F**	**NA**	**Neg**	**Neg**	**Neg**	**Neg**	**Neg**	**Neg**	**Neg**	**Neg**	**Neg**	**Neg**
Cathepsin K	Neg	Neg	Neg	Neg	Neg	NA	Neg	Neg	Neg	Neg	Neg	Neg	Neg	Neg	Neg	Neg
S100A1	60%	90%	90%	Neg	Neg	NA	90%	60%	80%	Neg	90%	90%	90%	Neg	80%	90%
BRAF	Neg	Neg	Neg	Neg	Neg	NA	Neg	Neg	Neg	Neg	Neg	Neg	Neg	Neg	Neg	Neg
**MUC1**	**30% F**	**70%**	**Neg**	**Neg**	**70%**	**NA**	**Neg**	**Neg**	**Neg**	**Neg**	**Neg**	**Neg**	**Neg**	**20% F**	**Neg**	**80%**
Ki67	1%	< 1%	5%	< 1%	< 1%	NA	5%	< 1%	< 1%	< 1%	< 1%	< 1%	2%	< 1%	2%	< 1%
WT1	Neg	NA	Neg	Neg	Neg	NA	Neg	Neg	Neg	Neg	Neg	Neg	Neg	Neg	Neg	Neg

Abbreviations: *E/OPRCC* Eosinophilic/oncocytic papillary renal cell carcinoma, *CK* Cytokeratin, *F* Focal, *Neg.* Negative, *NA* Not available, *CAIX* Carbonic anhydrase IX, *AMACR* Alpha-methylacyl-CoA racemase, *PV* Parvalbumin

### FISH and molecular pathology

**Fig. 5:**
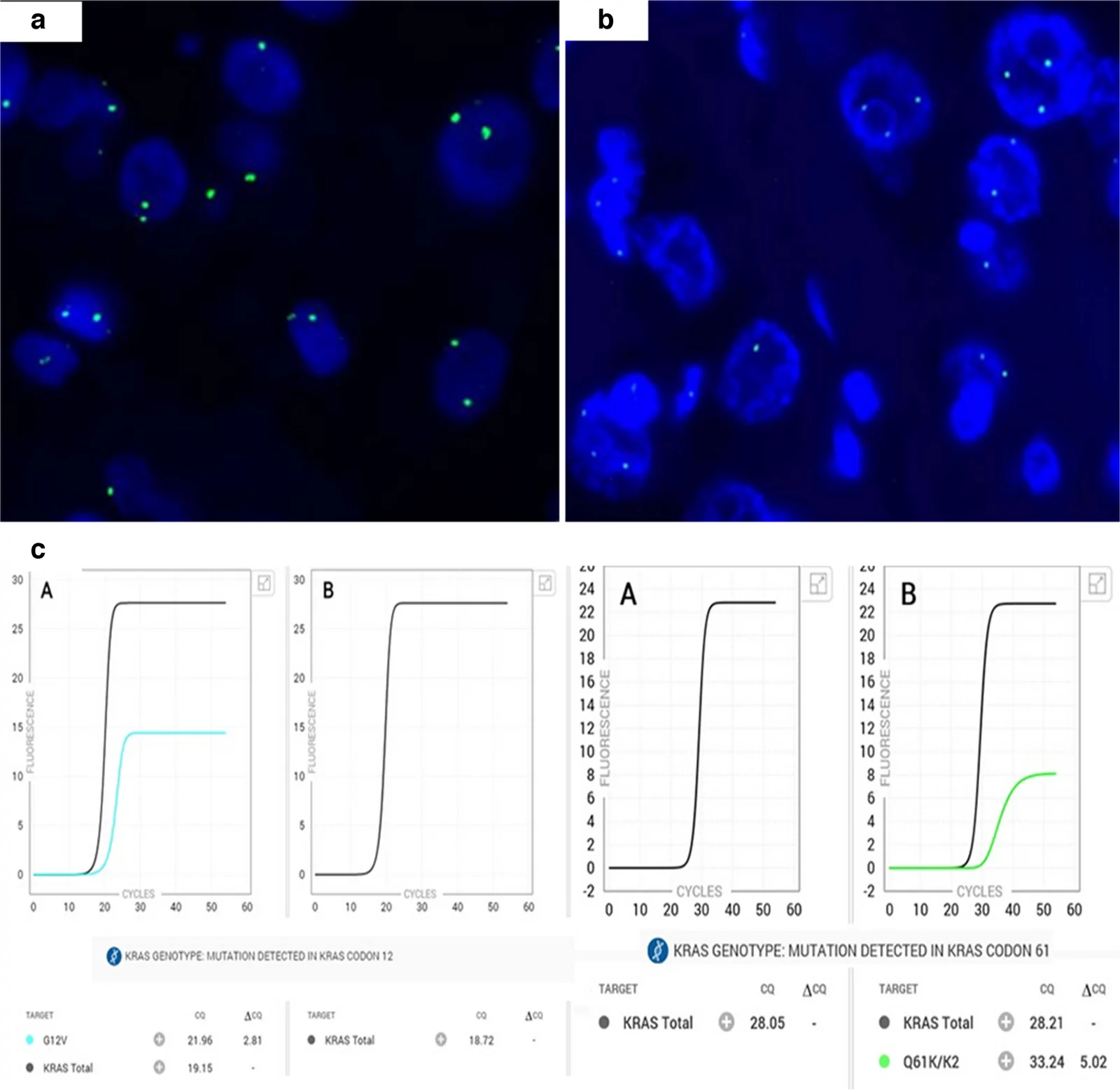
FISH for chromosome 17, revealing a disomic karyotype in PRNRP case 14 (**a**) and a trisomic karyotype in E/OPRCC case 12 (**b**). Molecular analysis revealed mutations of the *KRAS* gene in all the PRNRP tested. Representative curves from case 2 (p.G12V, A) and case 4 (p.Q61K, B) are shown (**c**)

At FISH analysis, no PRNRP showed chromosome 7 or 17 trisomy. A disomic chromosomal pattern (Fig. 5A), along with retention of the Y chromosome, was documented in all but two PRPRP samples with available material (82%, 9/11). These latter cases included one tumor with chromosome 7 monosomy (case 3, 60% split signals) and a PRNRP (case 10) displaying monosomy of both chromosome 7 (50% split signals) and 17 (60% split signals). The E/OPRCC group, by contrast, displayed a broader range of chromosomal abnormalities. One tumor showed trisomy 7 (7%), several others trisomy 17 (38%) (Fig. 5B), and one neoplasm carried trisomy of both chromosomes (7%). In addition, loss of the Y chromosome was identified in two tumors (33%). When the two diagnostic categories were compared, PRNRP showed a low rate of numerical cytogenetic alterations (18%, 2/11), whereas E/OPRCC exhibited abnormalities in roughly half of the evaluable cases (43%, 6/14). Some tumors in both groups could not be assessed because of limited tissue or technical artifacts and were therefore considered non‑informative.

Molecular analysis further highlighted the biological distinction between the two entities. All PRNRPs with available material harbored *KRAS* mutations (Fig. 5C), most commonly involving codon 12 in all but one sample (case 4, Q61K mutation at codon 61). Oppositely, nearly all E/OPRCCs were KRAS wild‑type, apart from one tumor (case 14) carrying an uncommon *KRAS* variant (p.A164P/T/V) differing from those observed in PRNRP. To note, in a few cases—PRNRPs 6 and 15 and E/OPRCC 2—molecular testing could not be performed because the material was insufficient or had been returned to the referring institution.

FISH and molecular findings of PRNRPs and E/OPRCC are listed in Table 5.

**Table 5:** FISH and molecular features of the PRNRPs and E/OPRCCs from the present cohort

Case n	Histotype	*KRAS* status	CEP 7 (n.)	CEP 17 (n.)	CEP Y
1	PRNRP	p.G12V	Disomic (2)	Disomic (2)	Present
2	PRNRP	p.G12V	Disomic (2)	Disomic (2)	Present
3	PRNRP	p.G12R	Monosomic (1)	Disomic (2)	Present
4	PRNRP	p.Q61K	NA	NA	NA
5	PRNRP	p.G12V	Disomic (2)	Disomic (2)	Present
6	PRNRP	NA	NA	NA	NA
7	PRNRP	p.G12D	Disomic (2)	Disomic (2)	Present
8	PRNRP	p.G12V	Disomic (2)	Disomic (2)	Present
9	PRNRP	p.G12D	Disomic (2)	Disomic (2)	Present
10	PRNRP	p.G12V	Monosomic (1)	Monosomic (1)	Present
11	PRNRP	p.G12V	NA	NA	Female
12	PRNRP	p.G12V	Disomic (2)	Disomic (2)	Female
13	PRNRP	p.G12D	Disomic (2)	Disomic (2)	Female
14	PRNRP	p.G12V	Disomic (2)	Disomic (2)	Present
15	PRNRP	NA	NA	NA	Female
1	E/OPRCC	WT	Disomic (2)	Disomic (2)	NA
2	E/OPRCC	NA	NA	NA	NA
3	E/OPRCC	WT	Disomic (2)	Disomic (2)	Present
4	E/OPRCC	WT	Disomic (2)	Trisomic (3)	NA
5	E/OPRCC	WT	Disomic (2)	Trisomic (3)	Female
6	E/OPRCC	WT	NA	NA	Present
7	E/OPRCC	WT	Trisomic (3)	Disomic (2)	Loss
8	E/OPRCC	WT	Disomic (2)	NA	NA
9	E/OPRCC	WT	Disomic (2)	Disomic (2)	Present
10	E/OPRCC	WT	Disomic (2)	Trisomic (3)	NA
11	E/OPRCC	WT	Disomic (2)	Disomic (2)	NA
12	E/OPRCC	WT	Disomic (2)	Trisomic (3)	NA
13	E/OPRCC	WT	Disomic (2)	Disomic (2)	Loss
14	E/OPRCC	p.A164P/T/V	Disomic (2)	Disomic (2)	Present
15	E/OPRCC	WT	Trisomic (3)	Trisomic (3)	NA
16	E/OPRCC	WT	Disomic (2)	Disomic (2)	NA

Abbreviations: *CEP* Centromere Enumeration Probes, *PRNRP* Papillary renal neoplasm with reverse polarity, *NA* Not available, *E/OPRCC* Eosinophilic/oncocytic papillary renal cell carcinoma, *WT* Wild type

### Literature review and statistical analysis

Since the first description in 2019[11], four hundred fifty-one PRNRPs, including the present study, have been reported. As for pathological features, available literature data indicate that almost 90% of PRNRP have an average diameter of less than 2 cm (mean 1.5 cm, range 0.02–9.5 cm) (Table S1). Moreover, hardly all tumors (99%) were low-grade according to ISUP/WHO criteria (G1–G2). None of the reported lesions showed necrosis or a conspicuous mitotic rate (Table S2). To date, none of the tumors described behaved aggressively, developing either local recurrences or distant metastases. Immunohistochemically, published cases have demonstrated constant and strong positivity for CK7 (96%), GATA3 (99%), PAX8 (97%), E‑cadherin (80%), EMA (99%), CK AE1/AE3 (100%), and CK34βE12 (81%). Oppositely, they generally failed to stain for AMACR (58% negative), CD117 (99% negative), CD10 (78% negative), and CAIX (95% negative) (Table S3). Trisomy of chromosomes 7 and/or 17 was documented in 16% of PRNRPs, while deletion of the Y chromosome was observed in 9% of cases (Table S4). As for molecular alterations, the wide majority (84%, 270/321) of the tumors tested carried *KRAS* mutations, involving, in order of frequency, the following hotspots: p.G12V (51%, 163/321), p.G12D (15%, 48/321), p.G12R (4%, 12/321), p.G12C (4%, 12/321), p.G12A/V/D (1%, 2/321), p.G13G (< 1% 1/321), and p.Q61K (< 1% 1/321) (Table S5).

Comparing PRNRP and E/OPRCC, they demonstrated clearly divergent clinical-pathological findings and molecular profiles. Morphologically, PRNRP were significantly smaller than E/OPRCC, with mean diameters of 1.5 cm and 3.4 cm, respectively (p = 0.0052). Their architecture was mostly papillary–cystic (82%), whereas E/OPRCC more often displayed a mixed papillary–tubular pattern (37%; p = 0.0951). This structural uniformity was matched by their low-grade nature: hardly any PRNRP was ISUP/WHO grade 1–2, in contrast to E/OPRCC, 56% of which were grade 3–4 (p < 0.0001). In this view, in contrast to PRNRP, our and other published E/OPRCC series document a low but still well-defined risk of an aggressive behavior, with 3–12% of the tumors developing either local recurrences or distant metastases[5, 19, 24, 25].

Additional histologic features further separated the two groups. Namely, psammoma bodies, almost absent in all PRNRP, appeared in 31% of E/OPRCC (p < 0.0001), and foamy macrophages were significantly more frequent in E/OPRCC (62% vs 17%; p = 0.0001), underscoring the broader morphologic variability of E/OPRCC (Table 6).

**Table 6:** Statistical comparison of clinical-pathological features between PRNRP (present series and published literature) and E/OPRCC (present series)

Parameter	PRNRP (present series)	E/OPRCC (present series)	PRNRP (literature)	*p*-value PRNRP vs E/OPRCC
Age (y.o.)				
Mean	69.6	67.1	63	0.4370
Range	52–86	54–79	25–86	
Sex				
Male	11/15 (73.3%)	15/16 (93.7%)	228/402 (56.6%)	**0.0031**
Female	4/15 (26.7%)	1/16 (6.3%)	174/402 (43.4%)	
Size (cm)				
Mean	1.42	3.41	1.5	**0.0052**
Range	0.4–3.2	0.4–10	0.02–9.5	
Architecture				
Papillary-cystic	15/15 (100%)	10/16 (62.5%)	267/327 (81.6%)	0.0951
Papillary-tubular	0/15 (0%)	6/16 (37.5%)	60/327 (18,4%)	
ISUP/WHO grading				
1–2 (low grade)	15/15 (100%)	7/16 (43.7%)	405/412 (98.3%)	** < 0.0001**
3–4 (high grade)	0/15 (0%)	9/16 (56.3%)	7/412 (1.7%)	
Tumoral necrosis				
Present	0/15 (0%)	2/16 (12.5%)	3/221 (1.3%)	0.0469
Absent	0/15 (100%)	14/16 (87.5%)	218/221 (98.7%)	
Foamy macrophages				
Present	2/15 (13.3%)	10/16 (62.5%)	47/274 (17.2%)	**0.0001**
Absent	13/15 (86.7%)	6/16 (37.5%)	227/274 (82.8%)	
Psammomatous bodies				
Present	0/15 (0%)	5/16 (31.2%)	2/208 (0.9%)	** < 0.0001**
Absent	15/15 (100%)	11/16 (68.8%)	206/208 (99.1%)	

Abbreviations: *PRNRP* Papillary renal neoplasm with reverse polarity, *E/OPRCC* Eosinophilic/oncocytic papillary renal cell carcinoma, *y.o.* years old, *NA* Not available

The bold indicates statistically significant *p*-values

The immunohistochemical profile of these two entities showed further significant distinctions, strengthening the notion that PRNRP and E/OPRCC represent biologically distinct entities rather than variants of a single tumor type (Table 7). In detail, PRNRPs consistently expressed CK7 (96% vs 40%; p < 0.0001) and GATA3 (99% vs 0%; p < 0.0001). In comparison to E/OPRCC, PRNRPs they also demonstrated significantly higher expression of EMA (99% vs 40%; p < 0.0001), CKAE1/AE3 (100% vs 47%; p = 0.0194), CK34βE12 (81% vs 20%; p < 0.0001), parvalbumin (64% vs 14%, p = 0.0168) and E‑cadherin (80% vs 7%; p < 0.0001). In contrast, E/OPRCC showed a profile more reminiscent of classic papillary renal cell carcinoma, with significantly higher expression of racemase/AMACR (p < 0.0001), vimentin (p < 0.0001), CD10 (p = 0.0062), and CD13 (p < 0.0001). MUC1, uniformly positive in PRNRP, was present in only a subset of E/OPRCC (33%, p = 0.0027).

**Table 7:** Statistical comparison of immunohistochemical, FISH, and molecular findings between PRNRP (present series and published literature) and E/OPRCC (present series)

Parameter	PRNRP (present series)	E/OPRCC (present series)	PRNRP (literature)	*p*-value PRNRP vs E/OPRCC
Immunohistochemistry				
CK7				
Positive	10/10 (100%)	6/15 (40%)	333/345 (96.5%)	** < 0.0001**
Negative	0/10 (0%)	9/15 (60%)	12/345 (3,5%)	
CAIX				
Positive	0/10 (0%)	0/15 (0%)	6/120 (5%)	1
Negative	10/10 (100%)	15/15 (100%)	114/120 (95%)	
GATA3				
Positive	12/12 (100%)	0/15 (0%)	363/365 (99.4%)	** < 0.0001**
Negative	0/12 (0%)	15/15 (100%)	2/365 (0.6%)	
PAX8				
Positive	8/8 (100%)	10/14 (71.4%)	182/187 (97.4%)	0.2536
Negative	0/8 (0%)	4/14 (28.6%)	5/187 (2.6%)	
E-cadherin				
Positive	5/8 (62.5%)	1/14 (7.1%)	44/55 (80%)	** < 0.0001**
Negative	3/8 (37.5%)	13/14 (92.9%)	11/55 (20%)	
EMA				
Positive	8/8 (100%)	6/15 (40%)	145/146 (99.3%)	** < 0.0001**
Negative	0/8 (0%)	9/15 (60%)	1/146 (0.7%)	
CD10				
Positive	2/8 (25%)	13/15 (86.7%)	54/245 (22.1%)	**0.0062**
Negative	6/8 (75%)	2/15 (13.3%)	191/245 (77.9%)	
CD13				
Positive	0/8 (0%)	13/15 (86.7%)	NA	** < 0.0001**
Negative	8/8 (100%)	2/15 (13.3%)	NA	
CD117				
Positive	0/9 (0%)	0/15 (0%)	1/177 (0.6%)	1
Negative	9/9 (100%)	15/15 (100%)	176/177 (99.4%)	
CKAE1/AE3				
Positive	8/8 (100%)	7/15 (46.7%)	23/23 (100%)	**0.0194**
Negative	0/8 (0%)	8/15 (53.3%)	0/23 (0%)	
CK34βE12				
Positive	7/8 (87.5%)	3/15 (20%)	83/103 (80.6%)	** < 0.0001**
Negative	1/8 (12.5%)	12/15 (80%)	20/103 (19.4%)	
PV				
Positive	7/11 (63.6%)	2/14 (14.3%)	NA	**0.0168**
Negative	4/11 (36.4%)	12/14 (85.7%)	NA	
AMACR				
Positive	4/9 (44.4%)	15/15 (100%)	150/360 (41.7%)	** < 0.0001**
Negative	5/9 (55.6%)	0/15 (0%)	210/360 (58.3%)	
Vimentin				
Positive	0/8 (0%)	10/15 (66.7%)	36/344 (10.5%)	** < 0.0001**
Negative	8/8 (100%)	5/15 (33.3%)	308/344 (89.5%)	
MUC1				
Positive	8/8 (100%)	5/15 (33.3%)	NA	**0.0027**
Negative	0/8 (0%)	10/15 (66.7%)	NA	
FISH				
Trisomic CEP 7/17	0/11 (0%)	6/14 (42.8%)	17/107 (15.8%)	**0.0262**
CEP Y loss	0/9 (0%)	2/6 (33.3%)	4/45 (8.9%)	0.1407
*KRAS* status				
Mutated	13/13 (100%)	1/15 (6.7%)	270/321 (84.1%)	** < 0.0001**
p.G12V	8/13 (61.5%)	NA	163/321 (50.8%)	NA
p.G12D	3/13 (23.1%)	NA	48/321 (14.9%)	NA
p.G12R	1/13 (7.7%)	NA	12/321 (3.8%)	NA
p.G12C	NA	NA	12/321 (3.8%)	NA
p.G12A/V/D	NA	NA	2/321 (0.6%)	NA
p.Q61K	1/13 (7.7%)	NA	1/321 (0.3%)	NA
p.A164P/T/V	NA	1/15 (6.7%)	NA	NA
p.G12X	NA	NA	7/321 (2.1%)	NA
p.G13G	NA	NA	1/321 (0.3%)	NA
Mutated NA	NA	NA	24/321 (7.5%)	NA
WT	0/13 (0%)	14/15 (93.3%)	51/321 (15.9%)	NA

Abbreviations: *PRNRP* Papillary renal neoplasm with reverse polarity, *E/OPRCC* Eosinophilic/oncocytic papillary renal cell carcinoma, *CK* Cytokeratin, *CAIX* Carbonic anhydrase IX, *NA* Not available, *PV* parvalbumin, *AMACR* Alpha-methylacyl-CoA racemase, *FISH* Fluorescence *in situ* hybridization, *CEP* Centromere Enumeration Probes, *WT* Wild type

The bold indicates statistically significant *p*-values

Cytogenetically, trisomy of chromosomes 7 and/or 17 by FISH or molecular analysis techniques (i.e., CGH, chromosomal microarray, or NGS) was significantly more common in E/OPRCC (43%) than in PRNRP (16%; *p* = 0.0262), aligning with the canonical genetic profile of papillary renal cell carcinoma. PRNRP, by contrast, largely retained a disomic status. From a molecular standpoint, striking differences in the *KRAS* status were documented between the two groups of neoplasms. The broad majority of analyzed PRNRP harbored activating *KRAS* mutations (270/321), whereas 93% of E/OPRCC were *KRAS* wild‑type (*p* < 0.0001). In this setting, the unique-mutated E/OPRCC from our series carried a non‑hotspot p.A164P/T/V variant, distinct from the codon 12 and 61 mutations typical of PRNRP.

## Discussion

Papillary renal neoplasm with reverse nuclear polarity (PRNRP) is a recently described renal cell tumor, morphologically characterized by thin and branching papillary structures lined by benign-looking oncocytic cuboidal cells with apically oriented nuclei. In this study, we have demonstrated that PRNRP represents a rare renal tumor with distinctive histopathological, immunophenotypic, cytogenetic, molecular, and prognostic features compared with E/OPRCC. As for the current WHO classification, PRNRP are considered a morphological pattern of the papillary renal cell carcinoma category[20], with whom they have been previously compared[18]. Nevertheless, to the best of our knowledge, this is the most extensive study to comprehensively match the characteristics of PRNRP specifically with E/OPRCC, representing their primary differential diagnosis, as these neoplasms can often share similar morphological features.

Firstly, our study confirms that PRNRP is a rare tumor: among 2110 renal cell neoplasms collected over a decade, it accounted for 0.5% of all tumors [26]. However, it may represent a consistent proportion of papillary renal neoplasms, reaching up to 17% in some recent series[27]. Regarding clinical variables such as sex and age, PRNRP does not show significant differences compared with E/OPRCC. Macroscopically, however, the two neoplasms differ significantly in size, with PRNRP exhibiting a smaller mean diameter (p = 0.0052), and color, particularly when E/OPRCC contains abundant foamy macrophages, which imparts a yellowish hue that is never observed in PRNRP.

More pronounced morphological divergences between these two entities emerged from our work. In detail, PRNRPs showed significant differences from E/OPRCC in terms of architecture (papillary‑cystic in PRNRP versus papillary‑tubular‑solid in E/OPRCC, p = 0.0951), nucleolar grade (uniformly low, G1/G2, in PRNRP versus high, G3/G4, in more than 50% of E/OPRCC, p < 0.0001), and the presence of foamy macrophages within the stromal core of papillae (not found in PRNRP but present in approximately 60% of E/OPRCC, p = 0.0001). Finally, the characteristic linear arrangement of round nuclei aligned toward the luminal apex of the neoplastic cells was never observed in E/OPRCC. Globally, such morphological features suggest a more aggressive biological potential for E/OPRCC than PRNRP. Although likely inferred by the improper inclusion of mimickers (i.e., FH-deficient renal cell carcinoma, TFE3/TFEB-altered renal cell carcinoma, and PRNRP themselves), our and other E/OPRCC series have documented local recurrences or metastases in a variable but still noteworthy proportion (3–12%) of them[5, 19, 24, 25]. These findings underscore the pivotal need to distinguish PRNRPs (virtually benign) from E/OPRCCs (potentially aggressive).

**Fig. 6:**
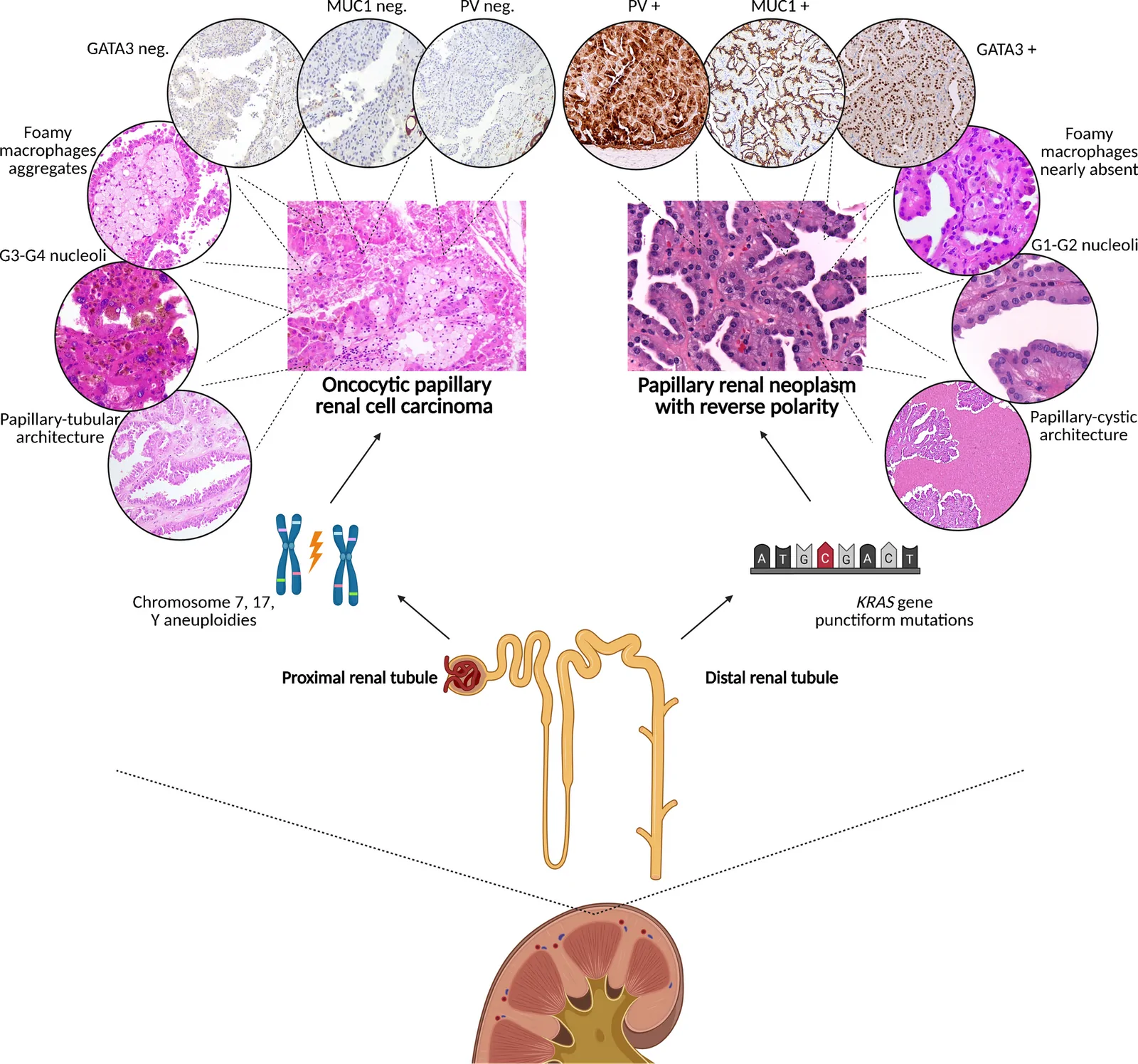
Schematic histogenic origin of E/OPRCC and PRNRP. Based on current knowledge, the former are thought to arise from the proximal renal tubule, driven by karyotype alterations involving chromosomes 7, 17, and Y. Conversely, point *KRAS* mutations in epithelial cells in the distal renal tubule are likely responsible for the development of PRNRP. This different origin is witnessed by the distinct immunophenotype of these neoplasms: E/OPRCC are usually positive for proximal tubular markers like CD10, CD13, and vimentin, but fail to stain for distal tubule markers, such as GATA3, parvalbumin, and CK34βE12. On the other hand, PRNRP tend to show an opposite profile (positivity for GATA3, parvalbumin, and CK34βE12, and negativity for CD10, CD13, and vimentin)

Immunohistochemical analysis revealed even greater discordances between PRNRP and E/OPRCC. PRNRP showed a consistent immunophenotype characterized by GATA3 positivity and vimentin negativity, in contrast to 0% (p < 0.0001) and 67% positivity (p < 0.0001), respectively, in E/OPRCC. Other markers with statistically significant differential expression included CK7 (p < 0.0001), AE1/AE3 (p = 0.0194), 34βE12 (p < 0.0001), E‑cadherin (p < 0.0001), EMA (p < 0.0001), parvalbumin (p = 0.0168), and MUC1 (p = 0.0027), which were more frequently positive in PRNRP, whereas racemase/AMACR (p < 0.0001), CD10 (p = 0.0062), and CD13 (p < 0.0001) were more commonly expressed in E/OPRCC. Therefore, PRNRP exhibits features shared with both more classic papillary renal cell carcinomas, particularly the former Type 1, such as CK7 positivity, and with common eosinophilic/oncocytic neoplasms (i.e., oncocytomas), including parvalbumin positivity and the near-complete absence of proximal markers CD13, CD10, and vimentin. From a histogenetic perspective, this immunophenotype ultimately supports a differentiation toward the distal nephron for PRNRP, in contrast to E/OPRCC (Fig. 6). In this view, parvalbumin is physiologically found in distal convoluted tubule cells of the nephron[28]. In renal tumors, its staining strongly states for a differentiation toward the distal tubule, as it is constantly positive in renal oncocytomas and chromophobe renal cell carcinomas[29–31]. Oppositely, it is typically negative in proximal tubule-arising histotypes, like clear cell renal cell carcinomas, papillary renal cell carcinomas, and clear cell papillary renal tumor[32–34]. GATA3 and CK34βE12 are two markers normally found in urothelium[35], distal convoluted tubules, and collecting system[36]. Expression of both these proteins by PRNRPs has been repeatedly reported and has led some Authors to claim a distal nephron differentiation for them[15, 16], supported by the molecular evidence of a distal nephron-enriched genetic signature[37]. Such a hypothesis is further strengthened in our series by common PRNRP parvalbumin positivity and their constant negativity for the proximal tubular markers CD10 and CD13, which are, instead, usually widely expressed by classic papillary renal cell carcinomas[38].

Cytogenetic and molecular data pointed out marked differences between PRNRP and E/OPRCC as well. Specifically, as for the former, no PRNRP showed trisomies, in contrast to approximately 40% of E/OPRCCs (p = 0.0262). According to published literature, classic papillary renal cell carcinomas carry a very high incidence of trisomy 7 (67%−100%) and 17 (94%−100%), and loss of the Y chromosome (100%)[39]. Conversely, available data on PRNRP are relatively inconsistent. While some studies, as in our series, report an almost complete absence of trisomies 7 and 17 and of Y-chromosome loss[40–42], others describe a significant frequency of these cytogenetic alterations, reaching up to 71% of the cases [11, 17]. As for other renal tumors for which FISH represents the gold-standard diagnosis (i.e., TFE3-rearranged and TFEB-altered renal cell carcinoma)[43], defining a broadly acknowledged cut-off is a challenging task. A putative explanation for these discrepancies may also lie in the different methodologies used to count immunofluorescent signals. Namely, like in our work, in some studies[40–42] signal interpretation was performed by comparing tumor nuclei with those of adjacent normal renal parenchyma[30], whereas others relied solely on signals observed within the tumor[11, 17]. Indeed, the comparison with non-neoplastic nuclei, along with the adoption of a higher (20% vs 10%)[41] positivity threshold than others, is certainly worth it in increasing the specificity of FISH findings, as confirmed by our previous experience in different renal cell tumors[44]. In this view, when molecular techniques apart from FISH were employed, such as CGH[41], chromosomal microarray[40], or NGS[14], no relevant copy number variation was documented in any of the tested chromosomal sequences.

Finally, both our and previously described PRNRP cases harbored a very high incidence of *KRAS* mutations (84%), in striking contrast to our E/OPRCC cohort (7%, p < 0.000001). The presence of *KRAS* mutations in PRNRP is particularly remarkable, given that alterations of this gene are found in only 1% of all renal cell carcinomas[37], making them exceedingly rare within renal neoplasia. Interestingly, KRAS-mutated neoplasms in other organs, such as the pancreas [45], lung[46], and colon[47], are associated with a poor prognosis, in contrast to the indolent biological behavior observed in PRNRP.

Overall, these statistically significant differences in size, architecture, grade, histologic features (psammoma bodies, foamy macrophages), immunoprofile (especially GATA3, CK7, CK34βE12, MUC1, CD13, CD10, racemase, vimentin, EMA, parvalbumin, and E‑cadherin), FISH findings (trisomies 7/17 and Y loss), and *KRAS* status strongly support the view that PRNRP and E/OPRCC represent two biologically and diagnostically distinct renal cell neoplasms rather than variations along a single spectrum. Taken together, the data from our study advocate the need to reconsider the current WHO nosologic classification of PRNRP[48], as suggested by other Authors[49]. In this view, certain morphologic and immunophenotypic features shared with eosinophilic/oncocytic distal nephron–derived tumors (oncocytoma and chromophobe renal cell carcinoma) might suggest including PRNRP within this subcategory. Nevertheless, the consistent presence of *KRAS* mutations in the wide majority of the cases, regardless of tumor size, could support the classification of PRNRP among molecularly defined renal neoplasms, even though these tumors are readily identifiable based on morphology and immunohistochemical features, in contrast to other entities currently regarded as “molecularly defined renal cell carcinomas”. However, *KRAS* mutation should not be regarded as a diagnostic hallmark of PRNRP, but rather as a desirable ancillary finding to be interpreted within the appropriate morphologic and immunophenotypic context. As demonstrated both in the present series and in the literature, E/OPRCCs, and more broadly PRCCs [37], may also harbor, rarely, *KRAS* gene mutations.

In conclusion, in the present work, we have demonstrated that PRNRP should be regarded as a group of neoplasms characterized by specific and definable features distinct from papillary renal cell carcinoma, especially from E/OPRCC, with which they are not infrequently misdiagnosed. Distinguishing these two tumor entities is pivotal because while PRNRPs are considered benign, E/OPRCCs harbor metastatic potential. Retaining PRNRP within the papillary carcinoma category solely based on architectural similarity and CK7 expression is, in our view, limiting and potentially misleading. Not only did all the obtained data show substantial differences between PRNRP and classic papillary renal cell carcinoma, but also between PRNRP and the E/OPRCC subgroup. These findings support a revision of the current WHO nosological classification of PRNRP within the papillary renal cell carcinoma category, advocating for its recognition as a distinct entity.

## Supplementary Information

Below is the link to the electronic supplementary material.

Supplementary file1 (DOCX 46 KB)

Supplementary file2 (DOCX 22 KB)

Supplementary file3 (DOCX 32 KB)

Supplementary file4 (DOCX 19 KB)

Supplementary file5 (DOCX 21 KB)

## Data Availability

All data generated or analysed during this study are included in this published article [and its supplementary information files].

## Declarations

All procedures involving human participants received institutional review board approval (Prog. 4136CESC) and were in accordance with the ethical standards of the institutional and/or national research committee and with the declaration of Helsinki.
