# Supplementary material for: Papillary renal neoplasm with reverse polarity: a novel entity for the next WHO classification? a clinical-pathological and molecular study of 15 cases, compared to eosinophilic/oncocytic papillary renal cell carcinoma
Source: Virchows Arch. 2026 Apr 29;489(3):631–51. doi: 10.1007/s00428-026-04536-9 (PMC13582226; doi:10.1007/s00428-026-04536-9)
Supplement: Supplementary file 1 [file 428_2026_4536_MOESM1_ESM.docx]

**Table S1.** Clinical-pathological features of published PRNRPs.

| **Case n.** | **Author** | **Age** | **Sex** | **Size/laterality** | **TNM stage** | **Surgery** | **Follow up (months)** | **Other findings** |
| --- | --- | --- | --- | --- | --- | --- | --- | --- |
| 1-15 | Al-Obaidy et al., 2019 | 50 | M | 2.5 cm/L | pT1aNxMx | PN | NA | NA |
|  |  | 79 | F | 1.2 cm/L | pT1aNxMx | RN | NA | ESRD |
|  |  | 75 | F | 1 cm/R | pT1aNxMx | PN | 44 NED | NA |
|  |  | 69 | M | 1 cm/R | pT1aNxMx | PN | 16 NED | NA |
|  |  | 80 | M | 1.5 cm/L | pT1aNxMx | PN | NA | NA |
|  |  | 76 | F | 1.5 cm/L | pT1aNxMx | Renal biopsy | 2 NED | NA |
|  |  | 77 | F | 1 cm/R | pT1aNxMx | RN | 30 NED | ESRD |
|  |  | 66 | M | 1 cm/R | pT1aNxMx | RN | 80 NED | NA |
|  |  | 58 | F | 1.1 cm/R | pT1aNxMx | RN | 1 NED | ESRD |
|  |  | 46 | F | 3 cm/L | pT1aNxMx | PN | 1 NED | NA |
|  |  | 54 | M | 1.8 cm/R | pT1aNxMx | PN | 67 NED | NA |
|  |  | 66 | M | 3 cm/R | pT1aNxMx | PN | 17 NED | NA |
|  |  | 50 | M | 1.1 cm/R | pT1aNxMx | PN | 22 NED | NA |
|  |  | 75 | M | 0.8 cm/R | pT1aNxMx | RN | 7 NED | ESRD + ACKD |
|  |  | 50 | M | 2.3 cm/R | pT1aNxMx | PN | 222 NED | NA |
| 16-45 | Kim et al., 2020* | 60.6 ± 8.2* | 11 F / 19 M* | 1.8 ± 0.9 cm* | 30 pT1aNxMx* | 29 PN,1 RN* | 54.6 ± 47.0*, 2/30 DOC, 28/30 NED | NA |
| 46-58 | Tong et al., 2020 | 56 | M | 2.7 cm/R | pT1aNxMx | 11 PN, 2 RN* | 5.37 NED | NA |
|  |  | 51 | M | 2.8 cm/L | pT1aNxMx |  | 5.7 NED | NA |
|  |  | 61 | M | 2.5 cm/L | pT1aNxMx |  | 17.23 NED | NA |
|  |  | 42 | M | 2 cm/R | pT1aNxMx |  | 22.37 NED | NA |
|  |  | 60 | M | 4.5 cm/L | pT1bNxMx |  | 36.53 NED | NA |
|  |  | 78 | M | 1 cm/R | pT1aNxMx |  | 38.2 NED | NA |
|  |  | 52 | F | 1.5 cm/L | pT1aNxMx |  | NA | NA |
|  |  | 59 | F | 3 cm/R | pT1aNxMx |  | NA | NA |
|  |  | 42 | M | 1.5 cm/L | pT1aNxMx |  | 35.5 NED | NA |
|  |  | 63 | M | 1.9 cm/R | pT1aNxMx |  | 57.54 NED | NA |
|  |  | 50 | F | 3.2 cm/R | pT1aNxMx |  | 24 NED | NA |
|  |  | 60 | M | 1.5 cm/L | pT1aNxMx |  | 23 NED | NA |
|  |  | 79 | F | 2.5 cm/R | pT1aNxMx |  | 41 NED | NA |
| 59-65 | Zhou et al., 2020 | 69 | F | 2.5 cm/L | pT1aNxMx | 5 PN, 2 RN* | 48 NED | NA |
|  |  | 48 | M | 2.2 cm/R | pT1aNxMx |  | 24 NED | NA |
|  |  | 65 | M | 8 cm/L | pT2aNxMx |  | 7 NED | Prostate cancer |
|  |  | 61 | M | 2.5 cm/R | pT1aNxMx |  | 3 NED | NA |
|  |  | 74 | F | 1.2 cm/R | pT1aNxMx |  | 50 NED | NA |
|  |  | 58 | F | 2.5 cm/R | pT1aNxMx |  | 65 NED | NA |
|  |  | 54 | M | 1.5 cm/R | pT1aNxMx |  | 3 NED | NA |
| 66 | Lee et al., 2020 | 61 | M | 1.2 cm/L | pT1aNxMx | RN | 8 NED | CCRCC |
| 67-76 | Chang et al., 2021 | 58 (46-84)* | 4F/4M* | 1.6 (1-4.3) cm/ 6 R/4 L* | pT1NxMx* | 5 PN, 5 RN* | 39 (4-101) NED* | 4/8 ESRD* |
| 77-95 | Kiyozawa et al., 2024 | 66,9 (47-82)* | 11F/8 M* | 1,9 (1-5) cm/NA* | pT1NxMx* | NA* | 96 NED* | 3/19 ESRD* |
| 96-97 | Lobo et al., 2021 | NA | NA | NA | NA | NA | NA | NA |
| 98 | Zhang et al., 2021 | 71 | M | 1.8 cm/L | pT1aNxMx | PN | NA | NA |
| 99-148 | Al-Obaidy et al., 2022 | 48 | F | 1.4 cm/R | pT1aNxMx | PN | 25 NED | NA |
|  |  | 45 | M | 1 cm/NA | pT1aNxMx | PN | 84 NED | NA |
|  |  | 52 | F | 0.16 cm/L | pT1aNxMx | RN | 20 NED | ESRD |
|  |  | 52 | F | 0.14 cm/R | pT1aNxMx | RN | 20 NED | ESRD |
|  |  | 42 | M | 0.07 cm/R | pT1aNxMx | RN | 160 NED | ESRD |
|  |  | 60 | M | 0.14 cm/L | pT1aNxMx | RN | 113 DOC | ESRD |
|  |  | 60 | M | 0.19 cm/L | pT1aNxMx | RN | 113 DOC | ESRD |
|  |  | 60 | M | 2.8 cm/L | pT1aNxMx | RN | 113 DOC | ESRD |
|  |  | 64 | M | 0.09 cm/R | pT1aNxMx | RN | 45 DOC | ESRD |
|  |  | 54 | M | 0.14 cm/R | pT1aNxMx | PN | 134 NED | NA |
|  |  | 63 | F | 0.11 cm/L | pT1aNxMx | RN | 114 NED | NA |
|  |  | 56 | M | 0.21 cm/R | pT1aNxMx | RN | 105 NED | NA |
|  |  | 65 | M | 0.1 cm/L | pT1aNxMx | PN | NA | NA |
|  |  | 75 | M | 0.06 cm/R | pT1aNxMx | RN | 7 NED | ESRD |
|  |  | 54 | F | 1 cm/R | pT1aNxMx | RN | 35 NED | ESRD |
|  |  | 77 | F | 0.21 cm/NA | pT1aNxMx | RN | 84 NED | NA |
|  |  | 70 | F | 0.12 cm/L | pT1aNxMx | RN | 87 NED | ESRD |
|  |  | 52 | M | 0.09 cm/R | pT1aNxMx | RN | 80 NED | ESRD |
|  |  | 76 | M | 0.28 cm/L | pT1aNxMx | RN | 87 NED | NA |
|  |  | 51 | F | 0.16 cm/NA | pT1aNxMx | RN | 60 NED | ESRD |
|  |  | 54 | F | 0.04 cm/R | pT1aNxMx | RN | 60 NED | ESRD |
|  |  | 77 | M | 0.06 cm/L | pT1aNxMx | PN | 5 DOC | NA |
|  |  | 56 | F | 1.1 cm/NA | pT1aNxMx | PN | 75 NED | NA |
|  |  | 84 | F | 0.08 cm/R | pT1aNxMx | RN | 24 NED | ESRD |
|  |  | 72 | F | 0.14 cm/L | pT1aNxMx | RN | 9 DOC | ESRD |
|  |  | 33 | F | 0.08 cm/L | pT1aNxMx | RN | 52 NED | ESRD |
|  |  | 73 | F | 0.09 cm/R | pT1aNxMx | RN | NA | ESRD |
|  |  | 55 | M | 0.06 cm/L | pT1aNxMx | RN | 56 NED | ESRD |
|  |  | 68 | F | 1 cm/L | pT1aNxMx | PN | 36 NED | NA |
|  |  | 58 | F | 0.11 cm/L | pT1aNxMx | PN | 45 NED | NA |
|  |  | 71 | F | 0.39 cm/R | pT1aNxMx | RN | NA | NA |
|  |  | 64 | F | 3 cm/R | pT1aNxMx | RN | NA | NA |
|  |  | NA | NA | 1.6 cm/NA | pT1aNxMx | PN | NA | NA |
|  |  | 72 | F | 0.9 cm/L | pT1aNxMx | PN | 24 NED | NA |
|  |  | 62 | F | 0.06 cm/R | pT1aNxMx | PN | 30 NED | NA |
|  |  | 63 | F | 0.15 cm/L | pT1aNxMx | PN | 7 NED | NA |
|  |  | 58 | M | 0.08 cm/L | pT1aNxMx | RN | 22 NED | ESRD |
|  |  | 72 | M | 0.09 cm/L | pT1aNxMx | RN | 30 NED | ESRD |
|  |  | 74 | M | 0.18 cm/L | pT1aNxMx | RN | 1 NED | ESRD |
|  |  | 71 | M | 0.06 cm/L | pT1aNxMx | RN | 10 NED | ESRD |
|  |  | 71 | M | 0.02 cm/R | pT1aNxMx | RN | 10 NED | ESRD |
|  |  | 79 | F | 1.6 cm/R | pT1aNxMx | PN | 13 NED | NA |
|  |  | 68 | F | 0.17 cm/R | pT1aNxMx | RN | 16 NED | ESRD |
|  |  | 68 | F | 0.9 cm/NA | pT1aNxMx | RN | 16 NED | ESRD |
|  |  | 71 | M | 0.15 cm/R | pT1aNxMx | RN | 1 NED | NA |
|  |  | 72 | F | 1 cm/L | pT1aNxMx | PN | 12 NED | ESRD |
|  |  | 73 | M | 0.43 cm/L | pT1aNxMx | RN | 3 DOC | NA |
|  |  | 58 | M | 0.9 cm/R | pT1aNxMx | PN | 15 NED | NA |
|  |  | 68 | M | 3 cm/L | pT1aNxMx | Renal biopsy | 4 NED | NA |
|  |  | 68 | F | 2.1 cm/R | pT1aNxMx | PN | 8 NED | NA |
| 149-155 | Wei et al., 2022 | 67 | F | 1 cm/NA | pT1aNxMx | NA | 23 NED | NA |
|  |  | 40 | F | 8.5 cm/NA | pT2aNxMx | NA | 141 NED | NA |
|  |  | 49 | M | 2 cm/NA | pT1aNxMx | NA | 110 NED | NA |
|  |  | 71 | M | 3.5 cm/NA | pT1aNxMx | NA | 33 NED | NA |
|  |  | 74 | F | 3.5 cm/NA | pT1aNxMx | NA | 20 NED | NA |
|  |  | 61 | M | 0.9 cm/NA | pT1aNxMx | NA | 12 NED | CCRCC |
|  |  | 69 | M | 2.3 cm/NA | pT1aNxMx | NA | 12 NED | NA |
| 156 | Wang et al., 2022 | 35 | M | 1.7 cm/R | pT1aNxMx | PN | 6 NED | NA |
| 157-176 | Liu et al., 2022 | 69 | F | 2.5 cm/L | pT1aNxMx | RN | 72 NED | NA |
|  |  | 48 | M | 2.2 cm/R | pT1aNxMx | PN | 48 NED | NA |
|  |  | 65 | M | 2.8 cm/L | pT1aNxMx | RN | 31 NED | NA |
|  |  | 61 | M | 2.5 cm/R | pT1aNxMx | PN | 27 NED | NA |
|  |  | 51 | M | 1.8 cm/R | pT1aNxMx | PN | 23 NED | NA |
|  |  | 41 | M | 1.8 cm/R | pT1aNxMx | PN | 17 NED | NA |
|  |  | 71 | M | 2 cm/L | pT1aNxMx | PN | 15 NED | NA |
|  |  | 62 | M | 2 cm/R | pT1aNxMx | PN | 14 NED | NA |
|  |  | 46 | M | 1.5 cm/R | pT1aNxMx | PN | 10 NED | NA |
|  |  | 77 | M | 1 cm/L | pT1aNxMx | RN | 8 NED | NA |
|  |  | 25 | M | 0.8 cm/L | pT1aNxMx | PN | 5 NED | NA |
|  |  | 58 | F | 2.5 cm/R | pT1aNxMx | PN | 89 NED | NA |
|  |  | 74 | F | 1.2 cm/R | pT1aNxMx | PN | 74 NED | NA |
|  |  | 54 | M | 1.5 cm/R | pT1aNxMx | PN | 27 NED | NA |
|  |  | 68 | F | 1.7 cm/R | pT1aNxMx | PN | 23 NED | NA |
|  |  | 51 | F | 1.2 cm/R | pT1aNxMx | PN | 20 NED | NA |
|  |  | 54 | M | 1.5 cm/R | pT1aNxMx | PN | 11 NED | NA |
|  |  | 70 | M | 6 cm/L | pT1bNxMx | PN | 33 NED | NA |
|  |  | 51 | F | 1 cm/R | pT1aNxMx | PN | 32 NED | NA |
|  |  | 67 | M | 1.8 cm/R | pT1aNxMx | PN | 25 NED | NA |
| 177-192 | Shen M. et al., 2022 | 76 | F | 2.5 cm/R | pT1aNxMx | NA | 3 NED |  |
|  |  | 80 | M | 2.8 cm/R | pT1aNxMx | NA | 3 NED | CCRCC |
|  |  | 67 | M | 9.5 cm/L | pT2aNxMx | NA | 9 NED |  |
|  |  | 79 | M | 0.4 cm/R | pT1aNxMx | NA | 1 NED |  |
|  |  | 54 | M | 2.1 cm/L | pT1aNxMx | NA | 4 NED |  |
|  |  | 67 | M | 1.9 cm/R | pT1aNxMx | NA | 8 NED |  |
|  |  | 55 | M | 4.5 cm/L | pT1bNxMx | NA | 16 NED |  |
|  |  | 57 | F | 1.2 cm/R | pT1aNxMx | NA | 39 NED |  |
|  |  | 47 | F | 2.6 cm/L | pT1aNxMx | NA | 62 NED |  |
|  |  | 50 | F | 1 cm/R | pT1aNxMx | NA | 15 NED |  |
|  |  | 76 | F | 1.2 cm/L | pT1aNxMx | NA | 14 NED |  |
|  |  | 70 | M | 6 cm/L | pT1bNxMx | NA | 7 NED |  |
|  |  | 73 | M | 1 cm/R | pT1aNxMx | NA | 42 NED |  |
|  |  | 70 | M | 1.5 cm/R | pT1aNxMx | NA | 4 NED |  |
|  |  | 68 | F | 1.7 cm/R | pT1aNxMx | NA | 11 NED | CCRCC |
|  |  | 51 | F | 1.2 cm/R | pT1aNxMx | NA | 8 NED |  |
| 193-235 | Kim B. et al., 2023* | 64 (31-81)* | 19F/24 M* | 1.57 cm/NA* | pT1NxMx* | NA* | 49 (1-176) NED* |  |
| 236-237 | Shao et al., 2024 | 53 | F | 1.5 cm/L | pT1aNxMx | PN | 11 NED |  |
|  |  | 55 | F | 2.5 cm/R | pT1aNxMx | PN | 7 NED |  |
| 238-246 | Han et al., 2024 | 36 | M | 1.5 cm/R | pT1aNxMx | PN | 64 NED |  |
|  |  | 39 | F | 1.8 cm/R | pT1aNxMx | PN | 52 NED |  |
|  |  | 68 | F | 1 cm/R | pT1aNxMx | PN | 46 NED |  |
|  |  | 71 | M | 2 cm/R | pT1aNxMx | PN | 45 NED | PRCC, prostatic adenocarcinoma |
|  |  | 72 | M | 3.7 cm/L | pT1aNxMx | RN | 37 NED |  |
|  |  | 67 | M | 3 cm/R | pT1aNxMx | PN | 31 NED |  |
|  |  | 71 | M | 2.7 cm/L | pT1aNxMx | PN | 19 NED |  |
|  |  | 63 | M | 2.2 cm/L | pT1aNxMx | PN | 18 NED |  |
|  |  | 64 | F | 1.6 cm/R | pT1bNxMx | PN | 12 NED |  |
| 247-256 | Nemours et al. 2024 | 75 | F | 4 cm/NA | pT1aNxMx | RN | 120 NED |  |
|  |  | 61 | M | 1.3 cm/NA | pT1aNxMx | PN | 4 NED |  |
|  |  | 70 | M | 1.8 cm/NA | pT1aNxMx | PN | 1 NED |  |
|  |  | 75 | F | 4 cm/NA | pT1aNxMx | RN | 7 NED |  |
|  |  | 73 | M | 1.7 cm/NA | pT1aNxMx | PN | 173 DOC |  |
|  |  | 69 | M | 0.5 cm/NA | pT1aNxMx | PN | 7 NED |  |
|  |  | 59 | F | 1.3 cm/NA | pT1aNxMx | RN | 168 DOC |  |
|  |  | 80 | F | 1 cm/NA | pT1aNxMx | RN | NA |  |
|  |  | 79 | F | 1.6 cm/NA | pT1aNxMx | RN | 13 NED |  |
|  |  | 45 | F | 4 cm/NA | pT1bNxMx | RN | NA |  |
| 257-264 | Castillo et al. 2024* | 64 (51-79)* | 4F/4M* | 1.3 cm/NA* | pT1aNxMx* | NA* | 24 (5-124) NED* |  |
| 265 | Kannan et al., 2025 | 52 | F | 2.8 cm/L | pT1aNxMx | PN | NED |  |
| 266 | Xing et al., 2025 | 66 | M | 3.5 cm/L | pT1aNxMx | PN | 22 NED |  |
| 267-268 | Gonda et al., 2025 | 71 | M | 3 cm/R | pT1aNxMx | PN | 18 NED |  |
|  |  | 74 | F | 2 cm/L | pT1aNxMx | PN | 96 NED |  |
| 269 | Gonzalez et al., 2025 | 59 | F | 2 cm/L | pT1aNxMx | PN | NA |  |
| 270-304 | Wang et al., 2025* | 60* | 9F/26M | 1.5 cm/NA* | pT1aNxMx* | NA | 4-124 NED* |  |
| 305-311 | Kwon et al., 2025 | 64 | M | 1.6 cm/NA | pT1aNxMx | NA | 182 NED |  |
|  |  | 64 | M | 2.5 cm/NA | pT1aNxMx | NA | 54 NED |  |
|  |  | 34 | F | 2.6 cm/NA | pT1aNxMx | NA | 126 NED |  |
|  |  | 74 | M | 2.2 cm/NA | pT1aNxMx | NA | 5 NED |  |
|  |  | 50 | F | 3.2 cm/NA | pT1aNxMx | NA | NA |  |
|  |  | 60 | M | 1.5 cm/NA | pT1aNxMx | NA | NA |  |
|  |  | 79 | F | 2.5 cm/NA | pT1aNxMx | NA | NA |  |
| 312-313 | Wu et al., 2025 | 49 | M | 2.1 cm/NA | NA | Renal biopsy | 60 NED |  |
|  |  | 64 | M | 1.1 cm/L | pT1aNxMx | PN | 172 NED |  |
| 314-339 | Wen et al., 2025* | 62* | 13F/13M | 2.2 cm/NA* | pT1NxMx* | 24PN/2RN* | 2-33 NED* |  |
| 340 | Castro et al., 2025 | 70 | M | 1.5 cm/L | NA | Renal biopsy | 8 NED | Lung adenocarcinoma |
| 341 | Vangapandu et al., 2025 | 65 | M | 0.5 cm/L | pT1aNxMx | RN | 4 NED |  |
| 342 | Chen et al., 2025 | 65 | M | 4.5 cm/R | pT1bNxMx | PN | 10 NED |  |
| 343 | Nithagon et al., 2025 | 70 | M | 1.7 cm/L | pT1aNxMx | PN | 9 NED |  |
| 344-353 | Park et al., 2024* | 56* | 2F/8M* | NA | NA | NA | NA |  |
| 354-358 | Sanmiguel et al., 2025* | NA | 4F/1M* | NA | pT1-2NxMx* | NA | NED |  |
| 359-436 | Lee et al., 2026* | 61 (31-78)* | 47F/40M* | 1.5 cm/NA* | pT1NxMx* | 74PN/3RN | 46 (0-237); 65 NED, 12 DOC* |  |
| 437-451 | Present study, 2026 | 52 | M | 1.2 cm/L | pT1aNxMx | PN | 3 NED |  |
|  |  | 79 | M | 2 cm/R | pT1aNxMx | PN | 9 NED |  |
|  |  | 71 | M | 2.2 cm/L | pT1aNxMx | PN | 72 NED |  |
|  |  | 69 | M | 0.7 cm/R | pT1aNxMx | PN | 124 NED |  |
|  |  | 71 | M | 2 cm/L | pT1aNxMx | PN | 58 NED | Renal angiomyolipoma |
|  |  | 79 | M | 3.2 cm/R | pT1aNxMx | PN | NA | PRCC |
|  |  | 77 | M | 0.8 cm/R | pT1aNxMx | RN | 44 NED | OPRCC, renal oncocytoma |
|  |  | 77 | M | 3 cm/L | pT1aNxMx | PN | 44 NED | OPRCC, renal oncocytoma |
|  |  | 62 | M | 0.4 cm/R | pT1aNxMx | RN | 58 NED | CCRCC lung metastases |
|  |  | 66 | M | 0.4 cm/L | pT1aNxMx | PN | 1 NED | CCRCC |
|  |  | 62 | F | 0.5 cm/R | pT1aNxMx | PN | 2 NED | OPRCC |
|  |  | 76 | F | 2.3 cm/R | pT1aNxMx | PN | NA |  |
|  |  | 66 | F | 1 cm/R | pT1aNxMx | PN | 21 NED | Borderline mucinous ovarian tumor |
|  |  | 52 | M | 1.1 cm/R | pT1aNxMx | PN | 23 NED |  |
|  |  | 86 | F | 0.5 cm/L | pT1aNxMx | RN | 29 NED |  |

*referring to the entire casuistry, data of single tumors not shown.

Abbreviations: F: female, M: male, R: right; L: left, NA: not available, NED: no evidence of disease, DOC: dead of other causes, RN: radical nephrectomy, PN: partial nephrectomy, CCRCC: clear cell renal cell carcinoma, ESRD, end-stage renal disease, ACKD, acquired cystic kidney disease, PRCC: papillary renal cell carcinoma, OPRCC: oncocytic papillary renal cell carcinoma.
