# Supplementary material for: Papillary renal neoplasm with reverse polarity: a novel entity for the next WHO classification? a clinical-pathological and molecular study of 15 cases, compared to eosinophilic/oncocytic papillary renal cell carcinoma
Source: Virchows Arch. 2026 Apr 29;489(3):631–51. doi: 10.1007/s00428-026-04536-9 (PMC13582226; doi:10.1007/s00428-026-04536-9)
Supplement: Supplementary file 2 [file 428_2026_4536_MOESM2_ESM.docx]

**Table S2.** Morphological features of published PRNRPs.

| **Case n.** | **Author** | **Architecture** | **ISUP/WHO grading** | **Pseudocapsule** | **Foamy macrophages** | **Necrosis** | **Hemosiderin pigment** | **Mitoses** | **Psammomatous bodies** | **Inflammatory infiltrate** |
| --- | --- | --- | --- | --- | --- | --- | --- | --- | --- | --- |
| 1-15 | Al-Obaidy et al., 2019 | Papillary with hyaline stroma (11/15); tubular (4/15) | 1-2 | 10/15 | 1/15 | Absent | Absent | Absent | Absent | NA |
| 16-45 | Kim et al., 2020 | Papillary with hyaline stroma (24/30); tubular (18/30) | 1 (20/30), 2 (6/30), 3 (4/30) | NA | 12/30 | Absent | NA | NA | NA | 22/30 |
| 46-58 | Tong et al., 2020 | Papillary with hyaline stroma (7/13) tubular (1/13) | 1-2 | 9/13 | 3/13 | Absent | 3/13 | Absent | Absent | NA |
| 59-65 | Zhou et al., 2020 | Papillary with hyaline stroma (7/7) | 1-2 | 7/7 | Absent | Absent | Absent | Absent | Absent | NA |
| 66 | Lee et al., 2020 | Papillary with hyaline stroma | 1 | Present | Present | Absent | NA | NA | NA | NA |
| 67-76 | Chang et al., 2021 | Papillary with hyaline stroma (10/10); tubular (6/10) | 1-2 | NA | 1/10 | Absent | 7/10 | Absent | Absent | 3/10 |
| 77-95 | Kiyozawa et al., 2024 | Papillary with hyaline stroma (19/19) | 1-2 | 10/19 | 3/19 | Absent | NA | NA | Absent | NA |
| 96-97 | Lobo et al., 2021 | Papillary with hyaline stroma (2/2) | 1-2 | NA | NA | NA | NA | NA | NA | NA |
| 98 | Zhang et al., 2021 | Papillary with hyaline stroma | 1-2 | Present | NA | NA | NA | NA | NA | NA |
| 99-148 | Al-Obaidy et al., 2022 | Papillary with hyaline stroma (50/50); papillary, edematous and sclerotic (3/50) | 1-2 | NA | NA | Absent | Absent | Absent | Absent | NA |
| 149-155 | Wei et al., 2022 | Papillary with hyaline stroma (7/7) | 1-2 | 6/7 | Absent | NA | Absent | NA | Absent | NA |
| 156 | Wang et al., 2022 | Papillary with hyaline stroma | NA | NA | NA | NA | NA | NA | NA | NA |
| 157-176 | Liu et al., 2022 | Papillary with hyaline stroma (20/20) | 1-2 | NA | 2/20 | Absent | NA | Absent | Absent | NA |
| 177-192 | Shen M. et al., 2022 | Papillary with hyaline stroma (11/16) | 1-2 | 1/7 | Absent | Absent | 3/16 | Absent | Absent | 2/16 |
| 193-235 | Kim B. et al., 2023 | Papillary with hyaline stroma (43/43) | 1-2 | NA | NA | NA | NA | NA | NA | NA |
| 236-237 | Shao et al., 2024 | Papillary with hyaline stroma (2/2) | NA | NA | NA | NA | NA | NA | NA | NA |
| 238-246 | Han et al., 2024 | Papillary with hyaline stroma (9/9) | 1 (9/9) | 0/9 | 3/9 (focal) | NA | 5/9 | NA | 1/9 | 2/9 |
| 247-256 | Nemours et al. 2024 | Papillary with hyaline stroma (10/10) | 1 (10/10) | 4/10 | NA | NA | NA | NA | NA | NA |
| 257-264 | Castillo et al. 2024 | Papillary with hyaline stroma | 1-2 (8/8) | NA | NA | NA | NA | NA | NA | NA |
| 265 | Kannan et al., 2025 | Papillary with hyaline stroma | 1 | NA | NA | Absent | NA | NA | NA | NA |
| 266 | Xing et al., 2025 | Papillary with hyaline stroma | 1 | NA | NA | NA | NA | NA | NA | NA |
| 267-268 | Gonda et al., 2025 | Papillary with hyaline stroma | 1 (2/2) | Present | NA | Absent | Absent | NA | Absent | NA |
| 269 | Gonzalez et al., 2025 | Papillary with hyaline stroma | 1 | NA | NA | Absent | NA | NA | NA | NA |
| 270-304 | Wang et al., 2025 | Papillary with hyaline stroma | 1-2 (35/35) | 19/35 | 5/35 | Absent | NA | NA | 1/35 | 2/35 |
| 305-311 | Kwon et al., 2025 | Papillary with hyaline stroma | 1 (7/7) | NA | NA | Absent | NA | Absent | NA | NA |
| 312-313 | Wu et al., 2025 | Papillary with hyaline stroma | 2 (1/1) | NA | NA | NA | NA | NA | NA | NA |
| 314-339 | Wen et al., 2025 | Papillary with hyaline stroma | NA | NA | NA | NA | NA | NA | NA | NA |
| 340 | Castro et al., 2025 | Papillary with hyaline stroma | 1 | NA | NA | Absent | NA | Absent | NA | NA |
| 341 | Vangapandu et al., 2025 | Papillary with hyaline stroma | 1 | NA | NA | NA | NA | NA | NA | NA |
| 342 | Chen et al., 2025 | Papillary with hyaline stroma | NA | NA | NA | NA | NA | NA | NA | NA |
| 343 | Nithagon et al., 2025 | Papillary with hyaline stroma | 1 | Present | NA | NA | NA | NA | NA | NA |
| 344-353 | Park et al., 2024 | Papillary with hyaline stroma | 1-2 (10/10) | NA | NA | NA | NA | NA | NA | NA |
| 354-358 | Sanmiguel et al., 2025 | Papillary with hyaline stroma | NA | NA | NA | NA | NA | NA | NA | NA |
| 359-436 | Lee et al., 2026 | Papillary with hyaline stroma | 1 (24/77), 2 (50/77), 3 (3/77) | 62/77 | 14/77 | 3/77 | 3/77 | NA | NA | 30/77 |
| 437-451 | Present study, 2026 | Papillary with hyaline stroma | 1-2 (15/15) | 13/15 | 2/15 | Absent | 1/15 | Absent | Absent | 7/15 |

Abbreviations: NA: not available.
