# Supplementary material for: Papillary renal neoplasm with reverse polarity: a novel entity for the next WHO classification? a clinical-pathological and molecular study of 15 cases, compared to eosinophilic/oncocytic papillary renal cell carcinoma
Source: Virchows Arch. 2026 Apr 29;489(3):631–51. doi: 10.1007/s00428-026-04536-9 (PMC13582226; doi:10.1007/s00428-026-04536-9)
Supplement: Supplementary file 3 [file 428_2026_4536_MOESM3_ESM.docx]

**Table S3.** Immunohistochemical findings of published PRNRPs.

| **Author** | **Result** | **CK 7** | **CK AE1/AE3** | **EMA** | **CD 10** | **CA IX** | **CK34βE12** | **CD 117** | **Vimentin** | **AMACR** | **GATA3** | **LICAM** | **E-cadherin** | **PAX8** |
| --- | --- | --- | --- | --- | --- | --- | --- | --- | --- | --- | --- | --- | --- | --- |
| Al-Obaidy et al., 2019 | Positive | 12/14 | 14/14 | 14/14 | 14/14 F | NA | NA | 0/14 | 0/14 | 4/14 | 14/14 | 8/14 | NA | NA |
|  | Negative | 2/14 | 0/14 | 0/14 | 0/14 | NA | NA | 14/14 | 14/14 | 10/14 | 0/14 | 6/14 | NA | NA |
| Kim et al., 2020 | Positive | 30/30 | NA | 30/30 | 0/30 | NA | NA | NA | 5/30 | 19/30 | NA | NA | 23/30 | NA |
|  | Negative | 0/30 | NA | 0/30 | 30/30 | NA | NA | NA | 25/30 | 11/30 | NA | NA | 7/30 | NA |
| Tong et al., 2020 | Positive | 10/10 | NA | 10/10 | 0/10 | NA | NA | NA | 0/10 | 4/10 | 10/10 | 7/10 | NA | 9/10 |
|  | Negative | 0/10 | NA | 0/10 | 10/10 | NA | NA | NA | 10/10 | 6/10 | 0/10 | 3/10 | NA | 1/10 |
| Zhou et al., 2020 | Positive | 7/7 | NA | 7/7 | 7/7 | NA | NA | 0/7 | 0/7 | 7/7 | 7/7 | NA | NA | NA |
|  | Negative | 0/7 | NA | 0/7 | 0/7 | NA | NA | 7/7 | 7/7 | 0/7 | 0/7 | NA | NA | NA |
| Lee et al., 2020 | Positive | 1/1 | NA | 1/1 | 0/1 | 0/1 | 1/1 | NA | 0/1 | 0/1 | 1/1 | NA | 1/1 | NA |
|  | Negative | 0/1 | NA | 0/1 | 1/1 | 1/1 | 0/1 | NA | 1/1 | 1/1 | 0/1 | NA | 0/1 | NA |
| Chang et al., 2021 | Positive | NA | NA | NA | NA | NA | NA | NA | NA | NA | 10/10 | NA | NA | NA |
|  | Negative | NA | NA | NA | NA | NA | NA | NA | NA | NA | 0/10 | NA | NA | NA |
| Kiyozawa et al., 2024 | Positive | 14/14 | NA | 14/14 | 0/14 | NA | NA | NA | NA | 8/14 | 14/14 | NA | NA | 14/14 |
|  | Negative | 0/14 | NA | 0/14 | 14/14 | NA | NA | NA | NA | 6/14 | 0/14 | NA | NA | 0/14 |
| Lobo et al., 2021 | Positive | NA | NA | NA | NA | NA | NA | NA | NA | NA | NA | NA | NA | NA |
|  | Negative | NA | NA | NA | NA | NA | NA | NA | NA | NA | NA | NA | NA | NA |
| Zhang et al., 2021 | Positive | 1/1 | NA | NA | 0/1 | 0/1 | NA | 0/1 | 0/1 | NA | 1/1 | NA | NA | 1/1 |
|  | Negative | 0/1 | NA | NA | 1/1 | 1/1 | NA | 1/1 | 1/1 | NA | 0/1 | NA | NA | 0/1 |
| Al-Obaidy et al., 2022 | Positive | NA | NA | NA | NA | NA | NA | NA | 0/45 | 14/45 | 45/45 | 45/45 | NA | NA |
|  | Negative | NA | NA | NA | NA | NA | NA | NA | 45/45 | 31/45 | 0/45 | 0/45 | NA | NA |
| Wei et al., 2022 | Positive | 7/7 | NA | NA | 3/7 F | NA | NA | 1/7 | 1/7 | 4/7 | 7/7 | NA | NA | NA |
|  | Negative | 0/7 | NA | NA | 4/7 | NA | NA | 6/7 | 6/7 | 3/7 | 0/7 | NA | NA | NA |
| Wang et al., 2022 | Positive | 1/1 | 1/1 | 1/1 | 0/1 | 0/1 | NA | 0/1 | 0/1 | 0/1 | 1/1 | NA | NA | 1/1 |
|  | Negative | 0/1 | 0/1 | 0/1 | 1/1 | 1/1 | NA | 1/1 | 1/1 | 1/1 | 0/1 | NA | NA | 0/1 |
| Liu et al., 2022 | Positive | 18/18 | NA | NA | NA | NA | NA | 0/18 | NA | NA | 18/18 | NA | NA | NA |
|  | Negative | 0/18 | NA | NA | NA | NA | NA | 18/18 | NA | NA | 0/18 | NA | NA | NA |
| Shen M. et al., 2022 | Positive | 16/16 | NA | NA | 3/16 | 0/16 | 12/16 | 0/16 | 1/16 | 11/16 | 16/16 | NA | 14/16 | 16/16 |
|  | Negative | 0/16 | NA | NA | 13/16 | 16/16 | 4/16 | 16/16 | 15/16 | 5/16 | 0/16 | NA | 2/16 | 0/16 |
| Kim B. et al., 2023 | Positive | 38/43 | NA | 43/43 | 21/43 | NA | NA | NA | 1/43 | 4/43 | 43/43 | 43/43 | NA | 43/43 |
|  | Negative | 5/43 | NA | 0/43 | 22/43 | NA | NA | NA | 42/43 | 39/43 | 0/43 | 0/43 | NA | 0/43 |
| Shao et al., 2024 | Positive | 2/2 | NA | NA | 0/1 | 0/1 | NA | 0/2 | 0/2 | 0/2 | 2/2 | NA | 1/1 | 1/1 |
|  | Negative | 0/2 | NA | NA | 1/1 | 1/1 | NA | 2/2 | 2/2 | 2/2 | 0/2 | NA | 0/1 | 0/1 |
| Han et al., 2024 | Positive | 7/9 | NA | NA | 4/9 | 6/9 | NA | 0/9 | 3/9 | 7/9 | 9/9 | NA | NA | NA |
|  | Negative | 2/9 | NA | NA | 5/9 | 3/9 | NA | 9/9 | 6/9 | 2/9 | 0/9 | NA | NA | NA |
| Nemours et al. 2024 | Positive | 10/10 | NA | NA | NA | NA | NA | NA | 0/10 | 9/10 | 10/10 | 7/10 | NA | NA |
|  | Negative | 0/10 | NA | NA | NA | NA | NA | NA | 10/10 | 1/10 | 0/10 | 3/10 | NA | NA |
| Castillo et al. 2024 | Positive | NA | NA | NA | NA | NA | NA | NA | NA | NA | NA | NA | NA | NA |
|  | Negative | NA | NA | NA | NA | NA | NA | NA | NA | NA | NA | NA | NA | NA |
| Kannan et al., 2025 | Positive | 1/1 | NA | NA | NA | NA | NA | NA | NA | 0/1 | 1/1 | NA | NA | NA |
|  | Negative | 0/1 | NA | NA | NA | NA | NA | NA | NA | 1/1 | 0/1 | NA | NA | NA |
| Xing et al., 2025 | Positive | 1/1 | NA | 1/1 | 0/1 | 0/1 | NA | 0/1 | 0/1 | 0/1 | 1/1 | NA | NA | 1/1 |
|  | Negative | 0/1 | NA | 0/1 | 1/1 | 1/1 | NA | 1/1 | 1/1 | 1/1 | 0/1 | NA | NA | 0/1 |
| Gonda et al., 2025 | Positive | 2/2 | NA | NA | NA | NA | NA | NA | 0/1 | NA | 2/2 | NA | NA | 1/1 |
|  | Negative | 0/2 | NA | NA | NA | NA | NA | NA | 1/1 | NA | 0/2 | NA | NA | 0/1 |
| Gonzalez et al., 2025 | Positive | 1/1 | NA | 1/1 | NA | 0/1 | NA | NA | 0/1 | 0/1 | 1/1 | NA | NA | NA |
|  | Negative | 0/1 | NA | 0/1 | NA | 1/1 | NA | NA | 1/1 | 1/1 | 0/1 | NA | NA | NA |
| Wang et al., 2025 | Positive | 35/35 | NA | NA | NA | NA | NA | NA | 6/34 | 12/34 | 35/35 | NA | NA | NA |
|  | Negative | 0/35 | NA | NA | NA | NA | NA | NA | 28/34 | 22/34 | 0/35 | NA | NA | NA |
| Kwon et al., 2025 | Positive | 4/4 | NA | NA | NA | NA | NA | 0/4 | 0/4 | NA | 4/4 | NA | NA | NA |
|  | Negative | 0/4 | NA | NA | NA | NA | NA | 4/4 | 4/4 | NA | 0/4 | NA | NA | NA |
| Wu et al., 2025 | Positive | 1/1 | NA | NA | NA | NA | NA | 0/1 | NA | NA | 2/2 | NA | NA | 1/2 |
|  | Negative | 0/1 | NA | NA | NA | NA | NA | 1/1 | NA | NA | 0/2 | NA | NA | 1/2 |
| Wen et al., 2025 | Positive | 25/26 | NA | 17/17 | 5/19 | NA | NA | 0/11 | 5/21 | 5/25 | 19/19 | NA | NA | 7/10 |
|  | Negative | 1/26 | NA | 0/17 | 14/19 | NA | NA | 11/11 | 16/21 | 20/25 | 0/19 | NA | NA | 3/10 |
| Castro et al., 2025 | Positive | 1/1 | NA | NA | NA | NA | NA | NA | NA | NA | 1/1 | NA | NA | 1/1 |
|  | Negative | 0/1 | NA | NA | NA | NA | NA | NA | NA | NA | 0/1 | NA | NA | 0/1 |
| Vangapandu et al., 2025 | Positive | 1/1 | NA | NA | NA | NA | NA | NA | NA | 0/1 | 1/1 | NA | NA | 1/1 |
|  | Negative | 0/1 | NA | NA | NA | NA | NA | NA | NA | 1/1 | 0/1 | NA | NA | 0/1 |
| Chen et al., 2025 | Positive | 1/1 | NA | NA | 0/1 | 0/1 | 1/1 | NA | 1/1 | 0/1 | 1/1 | NA | NA | NA |
|  | Negative | 0/1 | NA | NA | 1/1 | 1/1 | 0/1 | NA | 0/1 | 1/1 | 0/1 | NA | NA | NA |
| Nithagon et al., 2025 | Positive | 1/1 | NA | NA | NA | 0/1 | NA | 0/1 | NA | 0/1 | 1/1 | NA | NA | 1/1 |
|  | Negative | 0/1 | NA | NA | NA | 1/1 | NA | 1/1 | NA | 1/1 | 0/1 | NA | NA | 0/1 |
| Park et al., 2024 | Positive | NA | NA | NA | NA | NA | NA | NA | NA | NA | NA | NA | NA | NA |
|  | Negative | NA | NA | NA | NA | NA | NA | NA | NA | NA | NA | NA | NA | NA |
| Sanmiguel et al., 2025 | Positive | NA | NA | NA | NA | NA | NA | NA | NA | NA | NA | NA | NA | NA |
|  | Negative | NA | NA | NA | NA | NA | NA | NA | NA | NA | NA | NA | NA | NA |
| Lee et al., 2026 | Positive | 75/77 | NA | NA | 14/77 | 0/77 | 63/77 | 0/77 | 13/77 | 38/77 | 75/77 | NA | NA | 77/77 |
|  | Negative | 2/77 | NA | NA | 63/77 | 77/77 | 14/77 | 77/77 | 64/77 | 39/77 | 2/77 | NA | NA | 0/77 |
| Present study, 2026 | Positive | 10/10 | 8/8 | 7/8 | 2/8 | 0/10 | 6/8 | 0/9 | 0/8 | 4/9 | 12/12 | NA | 6/8 | 8/8 |
|  | Negative | 0/10 | 0/8 | 1/8 | 6/8 | 10/10 | 2/8 | 9/9 | 8/8 | 5/9 | 0/12 | NA | 2/8 | 0/8 |

Abbreviations: CK: cytokeratin, CAIX: carbonic anhydrase IX, AMACR: alpha-methylacyl-CoA racemase, F: focal, NA: not available.
