# Supplementary material for: Papillary renal neoplasm with reverse polarity: a novel entity for the next WHO classification? a clinical-pathological and molecular study of 15 cases, compared to eosinophilic/oncocytic papillary renal cell carcinoma
Source: Virchows Arch. 2026 Apr 29;489(3):631–51. doi: 10.1007/s00428-026-04536-9 (PMC13582226; doi:10.1007/s00428-026-04536-9)
Supplement: Supplementary file 4 [file 428_2026_4536_MOESM4_ESM.docx]

**Table S4.** Documented chromosomal alterations in published PRNRPs.

| **Author** | **Chromosomal alteration** | **N. of cases (%)** |
| --- | --- | --- |
| Al-Obaidy et al., 2019 | Trisomy of chromosomes 7/17 | 7/15 (46.6%) |
|  | Loss of chromosome Y | 1/7 (14.2%) |
| Kim et al., 2019 | Trisomy of chromosomes 7/17 | NA |
|  | Loss of chromosome Y | NA |
| Tong et al., 2020 | Trisomy of chromosomes 7/17 | NA |
|  | Loss of chromosome Y | NA |
| Zhou et al., 2020 | Trisomy of chromosomes 7/17 | 5/7 (71.4%) |
|  | Loss of chromosome Y | 1/3 (33.3%) |
| Lee et al., 2020 | Trisomy of chromosomes 7/17 | NA |
|  | Loss of chromosome Y | NA |
| Chang et al., 2021 | Trisomy of chromosomes 7/17 | NA |
|  | Loss of chromosome Y | NA |
| Kiyozawa et al., 2024 | Trisomy of chromosomes 7/17 | 0/15 (0%) |
|  | Loss of chromosome Y | 0/11 (0%) |
| Lobo et al., 2021 | Trisomy of chromosomes 7/17 | NA |
|  | Loss of chromosome Y | NA |
| Zhang et al., 2021 | Trisomy of chromosomes 7/17 | NA |
|  | Loss of chromosome Y | NA |
| Al-Obaidy et al., 2022 | Trisomy of chromosomes 7/17 | NA |
|  | Loss of chromosome Y | NA |
| Wei et al., 2022 | Trisomy of chromosomes 7/17 | 0/7 (0%) |
|  | Loss of chromosome Y | 0/7 (0%) |
| Wang et al., 2022 | Trisomy of chromosomes 7/17 | NA |
|  | Loss of chromosome Y | NA |
| Liu et al., 2022 | Trisomy of chromosomes 7/17 | NA |
|  | Loss of chromosome Y | NA |
| Shen M. et al., 2022 | Trisomy of chromosomes 7/17 | 1/15 (6.6%) |
|  | Loss of chromosome Y | 2/8 (25%) |
| Kim B. et al., 2023 | Trisomy of chromosomes 7/17 | NA |
|  | Loss of chromosome Y | NA |
| Shao et al., 2024 | Trisomy of chromosomes 7/17 | NA |
|  | Loss of chromosome Y | NA |
| Han et al., 2024 | Trisomy of chromosomes 7/17 | NA |
|  | Loss of chromosome Y | NA |
| Nemours et al. 2024 | Trisomy of chromosomes 7/17 | NA |
|  | Loss of chromosome Y | NA |
| Castillo et al. 2024 | Trisomy of chromosomes 7/17 | NA |
|  | Loss of chromosome Y | NA |
| Kannan et al., 2025 | Trisomy of chromosomes 7/17 | NA |
|  | Loss of chromosome Y | NA |
| Xing et al., 2025 | Trisomy of chromosomes 7/17 | NA |
|  | Loss of chromosome Y | NA |
| Gonda et al., 2025 | Trisomy of chromosomes 7/17 | NA |
|  | Loss of chromosome Y | NA |
| Gonzalez et al., 2025 | Trisomy of chromosomes 7/17 | NA |
|  | Loss of chromosome Y | NA |
| Wang et al., 2025 | Trisomy of chromosomes 7/17 | 4/16 (25%) |
|  | Loss of chromosome Y | NA |
| Kwon et al., 2025 | Trisomy of chromosomes 7/17 | 0/7 (0%) |
|  | Loss of chromosome Y | NA |
| Wu et al., 2025 | Trisomy of chromosomes 7/17 | NA |
|  | Loss of chromosome Y | NA |
| Wen et al., 2025 | Trisomy of chromosomes 7/17 | NA |
|  | Loss of chromosome Y | NA |
| Castro et al., 2025 | Trisomy of chromosomes 7/17 | NA |
|  | Loss of chromosome Y | NA |
| Vangapandu et al., 2025 | Trisomy of chromosomes 7/17 | NA |
|  | Loss of chromosome Y | NA |
| Chen et al., 2025 | Trisomy of chromosomes 7/17 | NA |
|  | Loss of chromosome Y | NA |
| Nithagon et al., 2025 | Trisomy of chromosomes 7/17 | NA |
|  | Loss of chromosome Y | NA |
| Park et al., 2024 | Trisomy of chromosomes 7/17 | NA |
|  | Loss of chromosome Y | NA |
| Sanmiguel et al., 2025 | Trisomy of chromosomes 7/17 | 0/5 (0%) |
|  | Loss of chromosome Y | NA |
| Lee et al., 2026 | Trisomy of chromosomes 7/17 | NA |
|  | Loss of chromosome Y | NA |
| Present study, 2026 | Trisomy of chromosomes 7/17 | 0/11 (0%) |
|  | Loss of chromosome Y | 0/9 (0%) |

Abbreviations: NA: not available.
