# Supplementary material for: Papillary renal neoplasm with reverse polarity: a novel entity for the next WHO classification? a clinical-pathological and molecular study of 15 cases, compared to eosinophilic/oncocytic papillary renal cell carcinoma
Source: Virchows Arch. 2026 Apr 29;489(3):631–51. doi: 10.1007/s00428-026-04536-9 (PMC13582226; doi:10.1007/s00428-026-04536-9)
Supplement: Supplementary file 5 [file 428_2026_4536_MOESM5_ESM.docx]

**Table S5.** *KRAS* gene mutational status in published PRNRPs.

| **Author** | **N. of cases (overall)** | **Mutation hotspot** | **N. of cases** |
| --- | --- | --- | --- |
| Al-Obaidy et al., 2019 | 7/8 mutated | p.G12V | 6/8 |
|  |  | p.G12R | 1/8 |
|  | 1/8 WT |  |  |
| Kim et al., 2020 | 25/30 mutated | p.G12V | 16/30 |
|  |  | p.G12D | 8/30 |
|  |  | p.G12R | 1/30 |
|  | 5/30 WT |  |  |
| Tong et al., 2020 | 10/12 mutated | p.G12V | 4/12 |
|  |  | p.G12D | 4/12 |
|  |  | p.G12R | 1/12 |
|  |  | p.G12C | 1/12 |
|  | 2/12 WT |  |  |
| Zhou et al., 2020 | NA | NA |  |
| Lee et al., 2020 | 1/1 mutated | p.G12V | 1/1 |
| Chang et al., 2021 | 9/10 mutated | p.G12V | 3/10 |
|  |  | p.G12D | 4/10 |
|  |  | p.G12R | 2/10 |
|  | 1/10 WT |  |  |
| Kiyozawa et al., 2024 | 14/19 mutated | p.G12V | 7/19 |
|  |  | p.G12D | 3/19 |
|  |  | p.G12R | 1/19 |
|  |  | p.G12C | 1/19 |
|  | 5/19 WT |  |  |
| Lobo et al., 2021 | NA | NA |  |
| Zhang et al., 2021 | NA | NA |  |
| Wei et al., 2022 | 4/7 mutated | p.G12D | 3/7 |
|  |  | p.G12C | 1/7 |
|  | 3/7 WT |  |  |
| Al-Obaidy et al., 2022 | 29/50 mutated | p.G12V | 22/50 |
|  |  | p.G12D | 2/50 |
|  |  | p.G12R | 2/50 |
|  |  | p.G12C | 2/50 |
|  |  | p.G12A/V/D | 1/50 |
|  | 21/50 WT |  |  |
| Wang et al., 2022 | 1/1 mutated | p.G12A | 1/1 |
| Liu et al., 2022 | 16/18 mutated | p.G12V | 10/18 |
|  |  | p.G12D | 2/18 |
|  |  | p.G12R | 1/18 |
|  |  | p.G12C | 2/18 |
|  | 2/18 WT |  |  |
| Shen M. et al., 2022 | 13/14 mutated | p.G12V | 7/17 |
|  |  | p.G12D | 4/14 |
|  |  | p.G12C | 2/14 |
|  | 1/14 WT |  |  |
| Kim B. et al., 2023 | NA | NA |  |
| Shao et al., 2024 | NA | NA |  |
| Han et al., 2024 | 7/9 mutated | p.G12V | 3/9 |
|  |  | p.G12D | 4/9 |
|  | 2/9 WT |  |  |
| Nemours et al. 2024 | 7/10 mutated | p.G12X | 7/10 |
|  | 3/10 WT |  |  |
| Castillo et al. 2024 | NA | NA |  |
| Kanna et al. 2025 | NA | NA |  |
| Xing et al., 2025 | NA | NA |  |
| Gonda et al., 2025 | NA | NA |  |
| Gonzalez et al., 2025 | NA | NA |  |
| Wang et al., 2025 | 23/28 mutated | NA |  |
|  | 5/28 WT |  |  |
| Kwon et al., 2025 | 7/7 mutated | p.G12V | 4/7 |
|  |  | p.G12D | 2/7 |
|  |  | p.G12R | 1/7 |
| Wu et al., 2025 | 1/1 mutated | p.G12V | 1/1 |
| Wen et al., 2025 | NA | NA |  |
| Castro et al., 2025 | 1/1 mutated | p.G12V | 1/1 |
| Vangapandu et al., 2025 | NA | NA |  |
| Chen et al., 2025 | 1/1 mutated | NA |  |
| Nithagon et al., 2025 | 1/1 mutated | p.G12C | 1/1 |
| Park et al., 2024 | NA | NA |  |
| Sanmiguel et al., 2025 | 5/5 mutated | p.G12V | 2/5 |
|  |  | p.G12D | 2/5 |
|  |  | p.G12R | 1/5 |
| Lee et al., 2026 | 75/75 mutated | p.G12V | 66/75 |
|  |  | p.G12D | 6/75 |
|  |  | p.G12C | 2/75 |
|  |  | p.G13G | 1/75 |
| Present study, 2026 | 13/13 mutated | p.G12V | 8 |
|  |  | p.G12D | 3 |
|  |  | p.G12R | 1 |
|  |  | p.Q61K | 1 |

Abbreviations: WT: wild type, NA: not available.
